# Supplementary material for: Protocol for the melatools skin self-monitoring trial: a phase II randomised controlled trial of an intervention for primary care patients at higher risk of melanoma
Source: BMJ Open. 2017 Nov 28;7(11):e017934. doi: 10.1136/bmjopen-2017-017934 (PMC5719271; doi:10.1136/bmjopen-2017-017934)
Supplement: Supplementary file 1 [file bmjopen-2017-017934supp001.pdf]

UNIVERSITY OF CAMBRIDGE
Public Health and Primary Care
The Primary Care Unit

MelaTools
improving early diagnosis

NHS
National Institute for Health Research

MelaTools Skin Self-Monitoring study for primary care patients at higher risk of melanoma

Consent Form

Names of Researchers: Fiona Walter, Katie Mills

Please initial **each** box

1. I confirm that I have read and understand the Participant Information Sheet Version 2, dated 24 June 2016 for the above study and I confirm that the study procedures and information have been explained to me. I have had the opportunity to ask questions and I am satisfied with the answers and explanations provided. ☐
2. I understand that my participation in this study is voluntary and that I am free to withdraw at any time, without giving a reason and without my medical care or legal rights being affected. ☐
3. I understand that sections of my medical notes or information related directly to my participation in this study may be looked at by responsible individuals from the sponsor, regulatory authorities and research personnel where it is relevant to my taking part in research. I give permission for these individuals to have access to my records, including follow-up up to 10 years. ☐
4. I understand that my GP will be informed of my participation in this study and sent details of the study. No medical advice regarding my skin will be issued by the study team and it is my responsibility to contact my GP if I become concerned about a skin change or mole. I understand that the study team will inform my GP if my questionnaire indicates that I am more worried about my health than others. ☐
5. I understand that the researchers in charge of this study may close the study, or stop my participation in it at any time without my consent. ☐
6. I have read and understood my responsibilities for the study. ☐
7. If I am in the group that is invited to download the smartphone App, I understand that the App is accessible to the public on online App stores and is installed onto my own device at my own risk. The study team does not take responsibility for any affect that the App may have on my device or for damage to my device whilst using the App. I am welcome to delete the App from my device at any time. ☐
8. If I am in the group that is invited to download the smartphone App, I understand that the information collected on the App is for my personal use only and the study team will never ask to see this information. ☐
9. I agree to participate in this study. ☐

Title	Name of participant	Signature	Date

Title	Name of person taking consent	Signature	Date

Thank you for completing this form. White copy for research team; yellow copy for participant, blue copy for GP practice.
