# Supplementary material for: Protocol for the melatools skin self-monitoring trial: a phase II randomised controlled trial of an intervention for primary care patients at higher risk of melanoma
Source: BMJ Open. 2017 Nov 28;7(11):e017934. doi: 10.1136/bmjopen-2017-017934 (PMC5719271; doi:10.1136/bmjopen-2017-017934)
Supplement: Author's manuscript [file bmjopen-2017-017934.draft_revisions.pdf]

BMJ Open is committed to open peer review. As part of this commitment we make the peer review history of every article we publish publicly available.

When an article is published we post the peer reviewers' comments and the authors' responses online. We also post the versions of the paper that were used during peer review. These are the versions that the peer review comments apply to.

The versions of the paper that follow are the versions that were submitted during the peer review process. They are not the versions of record or the final published versions. They should not be cited or distributed as the published version of this manuscript.

BMJ Open is an open access journal and the full, final, typeset and author-corrected version of record of the manuscript is available on our site with no access controls, subscription charges or pay-per-view fees (http://bmjopen.bmj.com).

If you have any questions on BMJ Open's open peer review process please email editorial.bmjopen@bmj.com

BMJ Open

# Protocol for the MelaTools Skin Self-Monitoring Trial: a phase II randomised controlled trial of an intervention for primary care patients at higher risk of melanoma



| | |
|---|---|
| Journal: | *BMJ Open* |
| Manuscript ID | bmjopen-2017-017934 |
| Article Type: | Protocol |
| Date Submitted by the Author: | 26-May-2017 |





| | |
|---|---|
| Complete List of Authors: | Mills, Katie; University of Cambridge, Public Health & Primary Care<br>Emery, Jon; University of Melbourne, General Practice and Primary Care Academic Centre<br>Lantaff, Rebecca; University of Cambridge, Public Health & Primary Care<br>Radford, Michael; University of Cambridge, Public Health & Primary Care<br>Pannebakker, Merel; University of Cambridge, Public Health & Primary Care<br>Hall, Per; Cambridge University Hospitals NHS Foundation Trust<br>Burrows, Nigel; Cambridge University Hospitals NHS Foundation Trust<br>Williams, Kate; University of Cambridge, Public Health & Primary Care<br>Saunders, Catherine; University of Cambridge, Cambridge Centre for Health Services Research<br>Murchie, Peter; University of Aberdeen, Division of Applied Health Science<br>Walter, Fiona; University of Cambridge, Dept of Public Health and Primary Care |





| | |
|---|---|
| <b>Primary Subject Heading</b>: | General practice / Family practice |
| Secondary Subject Heading: | Dermatology |
| Keywords: | melanoma, skin cancer, randomised controlled trial, risk-stratified, PRIMARY CARE |



SCHOLARONE™ Manuscripts

# Protocol for the MelaTools Skin Self-Monitoring Trial: a phase II randomised controlled trial of an intervention for primary care patients at higher risk of melanoma

Mills K[1], Emery JD[2,1], Lantaff R[1], Radford M[3], Pannebakker MM[1], Hall P[4], Burrows NP[4], Williams KM[1], Saunders CL[1], Murchie P[5], Walter FM[1,2]

[1] The Primary Care Unit, Department of Public Health and Primary Care, University of Cambridge, CB1 8SR, UK
[2] Department of General Practice and the Centre for Cancer Research, Faculty of Medicine, Dentistry and Health Science, Victorian Comprehensive Cancer Centre, University of Melbourne, Australia
[3] Tennyson House Surgery, Chelmsford CM1 4HW, UK
[4] Cambridge University Hospitals NHS Foundation Trust, Hills Road, Cambridge CB2 0QQ, UK
[5] Division of Applied Health Science, Centre of Academic Primary Care, University of Aberdeen, Polwarth Building, Foresterhill, Aberdeen AB25 2ZD, UK

**Correspondence to:** Katie Mills ko298@medschl.cam.ac.uk

**Key words:** melanoma, skin cancer, randomised controlled trial, risk-stratified, primary care

**Word Count**: 3630
2 figures, 1 table

For peer review only



For peer review only - http://bmjopen.bmj.com/site/about/guidelines.xhtml

**ABSTRACT (288/300 words)**

**Introduction**

Melanoma is the fifth commonest cancer in the UK. Incidence rates have quadrupled over the last 30 years and continue to rise, especially among younger people. As routine screening of the general population is not currently recommended in the UK, a focus on secondary prevention through early detection and prompt treatment in individuals at increased risk of melanoma could make an important contribution to improve melanoma outcomes. This paper describes the protocol for a phase II, multisite, randomised controlled trial, in the primary care setting, for patients at increased risk of melanoma. A skin self-monitoring smartphone 'App' was used to improve symptom appraisal and encourage help-seeking in primary care, thereby promoting early presentation with skin changes suspicious of melanoma.

**Methods and analysis**

We aim to recruit 200 participants from general practice waiting rooms in the East of England. Eligible patients are those identified at higher melanoma risk (using a real-time risk assessment tool), without a personal history of melanoma, aged 18 to 75 years. Participants will be invited to a primary care nurse consultation, and randomised to the Intervention group (standard written advice on skin cancer detection and sun protection, loading of a skin self-monitoring 'App' onto the participant's smartphone, and instructions on use including self-monitoring reminders) or Control group (standard written advice alone). The primary outcomes are consultation rates for changes to a pigmented skin lesion, and the patient interval (time from first noticing a skin change to consultation). Secondary outcomes include patient sun-protection behaviours, psychosocial outcomes, and measures of trial feasibility and acceptability.

**Ethics and dissemination**

NHS ethical approval has been obtained from Cambridgeshire and Hertfordshire research ethics committee (REC reference 16/EE/0248). The findings from the MelaTools SSM Trial will be disseminated widely through peer-reviewed publications and scientific conferences.

**Trial registration number:** ISCTRN registry, ISCTRN16061621


**Strengths and limitations of this study**

- The MelaTools SSM Trial is the first, in the UK or internationally, to risk stratify the primary care population in order to target an intervention to improve timeliness of melanoma detection.
- The study uses digital technology to encourage people at higher risk of melanoma to self-monitor their skin.
- Information from this trial will enable planning of a larger phase III trial to further assess the impact of the use of a smartphone App to encourage skin self-monitoring on clinical outcomes for melanoma.



For peer review only - http://bmjopen.bmj.com/site/about/guidelines.xhtml

## INTRODUCTION

Malignant melanoma is the leading cause of skin cancer deaths in the UK with 2,459 in 2014 (1). Melanoma skin cancer incidence has quadrupled over the last 30 years, and continues to rise (2). Although melanoma is more common with increasing age, around half of melanoma in the UK each year are diagnosed in people aged less than 65 (3, 4). Risk factors include fair skin, family history of melanoma, multiple naevi, and sun damage. Melanoma is associated with significant morbidity, and the thickness of the lesion at diagnosis is the most important prognostic factor: stage 1 disease has 5-year survival rates of over 95%, compared to less than 25% for stage 4 disease (5). The UK has lower 1- and 5-year melanoma survival rates than comparable countries in Europe (6). Diagnostic delays are thought to contribute to this, and there is evidence of avoidable delay (7). Evidence from the SCREEN project in Germany, conducted between 2003 and 2004, suggested that population screening may have an impact on melanoma incidence and 5-year mortality (8, 9). Although routine screening of the general population is not currently recommended worldwide, some countries (Australia, New Zealand, Germany, the Netherlands and the UK) recommend regular skin checks and/or self-examination for certain subsets of patients at increased risk of melanoma (10). Therefore, a focus on targeted population screening could make an important contribution to melanoma outcomes through early, timely detection and prompt treatment.

Patient pathways to presentation and management in primary care are key determinants in cancer outcomes. When compared with people diagnosed with other cancers, those diagnosed with melanoma have the second longest median time between first noticing a symptom and presenting to primary care (patient interval) (11, 12). The patient interval could potentially be reduced by providing patients with clear information on the signs and symptoms of melanoma and guidance on monitoring skin changes (13), through community campaigns such as the Be Clear on Cancer skin cancer campaign (14). More targeted approaches could focus on individuals at higher risk.

Smartphones offer considerable potential to promote earlier presentation by people with cancer, including melanoma (15). Most of the UK population now own a smartphone, and there are many smartphone applications ('Apps') focusing on health issues, presenting new opportunities for risk assessment, symptom appraisal, monitoring symptoms and signs over time, and cues to seek professional advice. Furthermore, up to 75% of melanomas are detected by people or their family/friends, rather than health care professionals (16). To maximise the effectiveness of skin self-monitoring (SSM) the person performing the examination should be able to identify skin changes and features of skin lesions which could indicate melanoma, yet recent studies have demonstrated that suspicious signs of skin cancer may not be widely known (17,13). Important international evidence has shown that it is possible to educate patients and the public: newly diagnosed Italian melanoma patients who performed self-skin examination were found to have thinner tumours (18), and Australian melanoma patients were found to adhere to medical advice on skin self-examination during follow-up care (19). A recent Scottish study has demonstrated melanoma patients are prepared to use digital technology to support them in conducting SSM during follow-up (20). We therefore set out to



For peer review only - http://bmjopen.bmj.com/site/about/guidelines.xhtml

explore using mobile technology for SSM among people at higher risk of melanoma in the primary care setting to encourage timely consultation for possible melanoma.

**Preliminary Phase I research**

Guided by the MRC framework on developing and evaluating complex interventions (21), three Phase I studies were conducted to: (i) assess the feasibility and acceptability of defining a population at higher risk of melanoma from UK general practice; (ii) review the availability of suitable smartphone SSM Apps, and (iii) pilot the intervention.

***(i) Assessing the feasibility and acceptability of defining a population at higher risk of melanoma from UK general practice***

The MelaTools-Q Study findings have already been reported (22). Briefly, 7,742 patients (86% of those approached) were recruited from the waiting rooms of 22 general practices in three UK regions: Eastern England, Northeast Scotland and North Wales. Using tablet computers, they completed an electronic questionnaire incorporating the Williams melanoma risk prediction model (23). The study showed that, after weighting to the age and sex distribution, the lower and upper quartile cut-offs used by Williams *et al.*, 25 and 34, would allow between 4% and 20% of the population to be identified as higher risk, and those groups would contain 30% and 60%, respectively of individuals who would develop melanoma. Therefore, real-time risk assessment for melanoma in UK primary care is both feasible and acceptable.

***(ii) Reviewing the availability of suitable smartphone SSM Apps***

Our initial review, conducted in July 2014 identified 39 smartphone Apps available from Apple and Android App online stores (24). However, when the same search strategy was re-run in September 2015, 8 new SSM Apps were identified, while 8 of the previously identified SSM Apps were no longer available. Among the 39 newly-identified SSM Apps, 11 were not available in both Apple and Android App stores; 10 did not include a monitoring function; 5 did not include photography of skin lesions; 5 had poor functionality; 2 were not available in England; 2 were classified as medical devices; one was under development, and one had no reminder feature, deemed vital for monitoring functionality. Two SSM Apps were considered potentially most suitable to include in the intervention (see Figure 1).

***(iii) Piloting the intervention***

Qualitative research, using focus groups and interviews, was conducted with individuals at increased risk of melanoma to provide in-depth understanding of consumer views on the usefulness and usability of the two selected SSM Apps. Eleven people at increased risk of melanoma were recruited for two focus groups from a local melanoma patient support group; several of their relatives also took part. People were also recruited in the waiting rooms of two suburban general practices and completed the Melatools-Q risk assessment tool (22). Five identified to be at increased risk of melanoma were invited to participate in two other focus groups. A total of 16 participants tested at least one of the SSM Apps. The participant characteristics are shown in Table 1.



For peer review only - http://bmjopen.bmj.com/site/about/guidelines.xhtml

The four focus groups were conducted by an experienced qualitative researcher (KM) and research assistant (RL), either at the recruiting general practice or university premises; each lasted approximately 90 minutes, was audio-recorded, and the recordings transcribed verbatim. Each participant was initially helped to load the two SSM Apps on their smartphones, and given time to familiarise themselves with their use. Thematic analysis (25) showed that, overall, participants in all four groups and at all strata of increased risk of melanoma felt that using a SSM App would be helpful and achievable. Many felt that using a SSM App would enhance their current skin self-monitoring habits: *"I look at the moles and I just keep an eye on them" (Male, above-population risk, group 3).* Many also felt that using a SSM App could help reduce their worry about developing skin cancer: *"cos my dad had cancer recently so [my mother] says we're at a higher risk, so she goes on about that" (Female, above-population risk, group 4).* Some felt that using a SSM App would guide their help-seeking behaviour for skin changes or moles: *"It's that knowledge, if it's, you know, three months, whatever it is, if you can actually say look there is a visible change then you're getting a bit of information that they probably could do with…you've got physical evidence, and then the confidence to go to…" (Male, above-population risk, group 4).* Several also commented on additional features on smartphones which might help prevent development of future melanomas: *"I've got a UV index on my phone so I can always check what that is, so putting sun cream on." (Male, high risk, group 2).*

The five participants recruited via primary care were invited to complete a diary one and two months later, and then participate in a telephone interview after the third month. Diary records and thematic analysis of the interviews (as above) confirmed that participants used many features of both SSM Apps, including: taking photographs, mapping these to body parts, comparing photographs over time, and using the analysis function (available in only one of the Apps). There was unanimous agreement between participants about the App which was easier to use (MySkinPal), and most reported that they would continue to use it after the study, particularly as they found the App's notifications the most efficient method to prompt them to complete future skin self-monitoring. Despite this, several also reported that they found taking photographs quite challenging, and that they had to recruit a family member to assist them in taking photographs to compare skin changes over time. Furthermore, while regularly self-monitoring their skin during the study, most participants reported worrying about skin cancer 'sometimes', seeking further information about skin changes or moles via the internet, speaking to friends and family, and visiting their general practice.

In conclusion, this preliminary Phase I work enabled consumer choice of the more user-friendly SSM App, resulted in modifications to the language used in the trial consultation, and led to the development of the MelaTools SSM Trial. The trial aims to assess the effect of using a SSM App compared with standard information about detecting skin cancer on consultation rates and the patient interval (the time from first noticing a skin change/mole to consultation with a primary care healthcare professional) among patients at increased risk of melanoma, identified via primary care. In addition, to obtain preliminary estimates of effect across a range of



For peer review only - http://bmjopen.bmj.com/site/about/guidelines.xhtml

outcome measures to inform selection of the primary outcome for a definitive phase III trial.

## METHODS AND ANALYSIS

### Study design and setting

The phase II trial is a multi-site randomised controlled trial (Figure 2). Those who meet the eligibility criteria and consent to participate are randomised 1:1 into either the control or intervention group. Randomisation is being performed using an online system provided by the Clinical Trials Unit based at Kings College London.

### Study participants

***Eligibility criteria***

Eligible participants are individuals aged between 18 and 75 years who own a smartphone (Apple or Android), and, on completion of the Melatools-Q risk assessment tool (22), are found to be at increased risk of melanoma. Participants are able to read and write English and to give informed consent.

***Exclusion criteria***

Exclusion criteria are previous diagnosis of melanoma, severe psychiatric or cognitive disorders, or a physical disorder severe enough to inhibit the use of a smartphone.

***Participant recruitment***

Potentially eligible individuals are approached by research nurses or research assistants in GP waiting areas at different times of the day and different days of the week, in order to ensure that a broad range of ages, gender, and educational level are approached. Those meeting initial screening criteria are given a tablet computer that includes a short version of the participant information sheet and an online consent form agreeing to have their risk assessed and be considered for the trial. They complete the MelaTools Q study risk assessment tool (22) which stratifies respondents into 'population risk' (majority) and 'increased risk', using a cut-off score in the Williams model of 25. People at 'population risk' are thanked and given the Cancer Research UK leaflets on risk factors of melanoma and melanoma detection advice. People at increased risk are invited to participate in the trial, and given the full-length trial Participant Information Sheet and an appointment time within 14 days for the primary care nurse trial consultation. Individuals not wishing to take part are asked their reason for declining.

***Control and Intervention groups and randomisation***

Informed written consent is obtained from all participants at the start of the trial consultation. After completion of a baseline questionnaire, the trial nurse guides the participant through the Cancer Research UK leaflets on "Be SunSmart cut your cancer risk" and "Skin cancer - How to spot the signs and symptoms". Online randomisation is then performed: those randomised to the control group are thanked for their participation and given the Cancer Research UK leaflets to take home.

***Intervention group***



For peer review only - http://bmjopen.bmj.com/site/about/guidelines.xhtml

Participants randomised to the intervention group are assisted with loading the skin self-monitoring App onto their smartphone by the trial nurse, who continues by giving instructions on its use, supported by written instruction sheets. The core functions of the App, MySkinPal, include: full body scan, body diagrams, photo source and browser, information on the signs of melanoma, regular reminders, ability to select moles to share with a doctor, and motivation to use including awards/achievements. The nurse ensures the App's monthly reminder notification is switched on to prompt each participant regularly. Finally, each intervention participant is given an Apple Apps store/Google Play voucher (up to £5) to pay for the App.

**Outcomes and measures**

The primary outcomes of this trial are *consultation rates* for pigmented skin lesions, and the *patient interval. Consultation rates* for changes to an existing pigmented skin lesion or concerns about a new pigmented skin lesion for the 12 months prior to the trial and the 12 months after the trial consultation will be collected via searching general practice electronic medical records using a clinical notes audit for every participant. The *patient interval* is the time from first noticing a skin change to first consultation. These data will be collected monthly by searches of general practice electronic medical records for all skin changes or pigmented skin lesions presented to their GP or practice nurse during the 12 months following the trial consultation. When a participant consults about a pigmented skin lesion, they receive a Skin Questionnaire to complete. This skin questionnaire uses the Symptom Study instrument modified for melanoma symptoms; a participant-completed questionnaire collecting data on symptoms and their duration prior to consultation, (25-27).

Secondary measures and outcomes (measured at baseline, 6- and 12-months) include:

(1) *Demographics and clinical variables:* age, gender, marital status, postcode, highest education level, occupation, past history of skin cancer (melanoma, Squamous Cell Carcinoma, Basal Cell Carcinoma), skin and hair type (density of freckles on arms before age 20, natural hair colour at age 15, number of severe sunburns aged 2-18), number of raised moles on both arms, measured at baseline only (22,23), collected as part of the baseline eligibility assessment;

(2) *Sun protection habits scale*: developed by Glanz et al in the US for a multicomponent skin cancer prevention programme, this comprises 5 items measured using a 4 point Likert scale, and relating to use of sun protection, sun and sunbed habits, and episodes of sunburn in the previous year (29), as we hypothesise that the App reminders will not only prompt people but also reinforce the messages on sun protection;

(3) *Skin Self-Examination benefits and barriers scale*: validated by Manne and Lessin in the US among melanoma survivors, and developed from previous work on mammography and family members of patients with colorectal cancer (30). The benefits scale has seven items (α=.71) and the barriers scale has ten items (α=.74);



For peer review only - http://bmjopen.bmj.com/site/about/guidelines.xhtml

(4) *Melanoma Worry Scale (MWS):* validated by Moye et al in the US (31), an adapted from the Breast Cancer Worry Scale (32), this measure comprises four items, scored 1 to 4, with possible scores ranging from 4 to 17, and higher scores indicating higher levels of worry;

(5) *Perceived Melanoma Risk*: drawn from Manne and Lessin's measures (30), these two items have been widely used for melanoma and other cancer risk assessments to assess estimated percent risk of developing melanoma, and perceived risk compared with a person of the same age (relative risk);

(6) *Self-Efficacy for consulting without delay*: A 10-item self-completed scale summed to score 10-100, was used in a primary care trial for lung symptoms, and showed good internal reliability (Cronbach α =0.85) (33, 34). It has been adapted for this trial, and reduced to 8 items;

(7) *Hospital anxiety and depression scale (HADS):* This 14-item self-completed scale has been widely used to measure distress and has been extensively validated and shown to perform well in a wide range of populations (mean Cronbach α = 0.82; sensitivity and specificity 0.80) (35);

(8) *SF-12 Quality of life scale*: a 12-item version of the SF-36 that is widely used and validated to measure functional health and well-being (36).

Other outcome measures include:

(9) *Trial feasibility and acceptability*, including: data on patient recruitment and attrition rates, reasons for attrition, response rates to Skin Symptom Questionnaires for measuring the patient interval to inform decisions about a future phase III trial.

(10) *Melanoma incidence across participating practices*, to contextualise trial findings and after 5 years. This will be identified through GP electronic medical records and the National Cancer Registration Service.

**Measurement timings and study end points**

At the trial consultation all participants will be fully informed about trial follow-up procedures. The participant-reported measures (numbers 1-8 above) are completed in person at baseline, and online at follow-up 1 (6 months) and follow-up 2 (12 months), with the exception of the skin symptom questionnaire as already described, which is sent to participants by postal mail within a month of any consultation for skin changes or pigmented skin lesions. After trial completion, a final audit of the GP electronic medical records will be run to identify all skin consultations for the 12 months during the trial as well as the previous 12 months. Melanoma incidence findings will also be collected at the end of the 12 month follow up period and at 5 years. These data will not be collected for participants who formally withdraw from the trial.

**Participant data and study management**

All participants are allocated a unique identifying code. Melanoma risk assessment data, baseline questionnaire, and follow up questionnaires data are stored on a custom built database on a secure server hosted by the Outcome Registry Intervention and Operation Network (ORION), Clinical Neurosciences department at the University of Cambridge.



For peer review only - http://bmjopen.bmj.com/site/about/guidelines.xhtml

**Sample size consideration**

Based on our recent MelaTools Q study with an identical screening step (response rate of 86%), we anticipate that we will need to approach approximately 2,000 people from 10 general practice waiting rooms for about 1,600 to complete the Melatools Q risk assessment tool (24). About 25%, 400 people, will be identified as increased risk and be eligible to participate in the trial. Based on previous research we would expect approximately 50% of these to attend their trial consultation and undergo randomisation in order to reach our target of 200 participants.

We will use information on recruitment, attrition, and data completeness, and preliminary estimates of effect sizes where appropriate, to inform the sample size calculation for a future phase III trial.

**Statistical analysis**

All randomised patients will be considered eligible for inclusion in the analysis in accordance with the intention-to-treat analysis principle. As this is a phase II trial, fully describing and characterising the extent and nature of the missing data is an important part of the analysis. For the outcome analysis, appropriate methods for dealing with missing endpoint data will be detailed in the statistical analysis plan and be informed by a blinded review of the data. The baseline characteristics of the two arms will be described using summary statistics. Possible consent bias will be assessed by comparing demographic and clinical variables of participants against those who declined participation, and possible differential attrition will be assessed by comparing baseline characteristics of those who withdraw or die against those who remain in the trial. These comparisons will be performed using a two sample t-test (or non-parametric equivalent) for continuous variables and chi-square test for categorical variables.

The primary analysis will be a comparison between the two groups on the consultation rate for skin changes/pigmented skin lesion using a Poisson regression model. Patient Interval is expected to be right skewed (37). We will explore the nature and extent of the skewness and will consider it as a continuous variable in the analysis, but may explore other approaches, including transformation, categorisation, or methods for statistical inference based on bootstrap resampling, as appropriate. Comparisons between groups on continuous secondary endpoints will be undertaken using a linear model that includes the baseline value where applicable. Comparisons between groups on binary secondary endpoints will be performed using logistic regression. The analyses performed on the primary and secondary endpoints will be repeated adjusting for additional baseline covariates as part of a sensitivity analysis. Point estimates of the intervention effect will be presented with 95% confidence intervals and two-sided p-values. Unadjusted p-values from secondary analyses will be interpreted in proper context and be clearly labelled.

**ETHICS AND DISSEMINATION**

**Ethical approval**

Ethical approval for the MelaTools SSM trial was granted on 11th July 2016 by the Cambridgeshire and Hertfordshire NRES research ethics committee (reference



For peer review only - http://bmjopen.bmj.com/site/about/guidelines.xhtml

number 16/EE/0248). Health Research Authority approval (HRA) was granted on 22nd July 2016. R&D approvals have been obtained from the relevant Trusts. All substantial amendments are approved by the NHS Research Ethics committee responsible for the trial, in addition to approval by HRA and NHS R&D. Investigators are kept up to date with relevant changes via regular management group meetings.

**Data monitoring**

Due to the non-medicinal and low-risk nature of the trial a data monitoring committee will not be needed. The trial steering committee (the CI, collaborators and researchers, statistician and PPIE representatives) will meet 6 monthly from the start of the trial and will monitor trial progress, approve a data analysis plan, and will ensure the trial runs in accordance with the protocol and applicable standard operating procedures. The CI will take responsibility for data monitoring and ethics, and will be responsible for communicating important protocol modifications to relevant parties.

**Dissemination**

Twelve-month follow-up will complete in January 2018. We will submit the findings of our research for publication in highly cited and open access peer-reviewed journals. These findings will also be presented at national and international conferences. We will also provide a summary of our findings on the MelaTools programme website MelaTools.org.

For peer review only

**Acknowledgements**

The authors are grateful to the patients and general practices who contributed to the pilot study. The authors would also like to thank the MelaTools Steering Committee for their scientific support and comments throughout the design and set-up of the trial: Pippa Corrie, Katharine Acland, Juliet Usher-Smith, Ed Wilson, Margaret Johnson, Simon Rodwell and Patricia Fairbrother. This study is supported by the UK Clinical Research Collaboration-registered King's Clinical Trials Unit at King's Health Partners, which is part-funded by the NIHR Biomedical Research Centre for Mental Health at South London and Maudsley NHS Foundation Trust and King's College London and the NIHR Evaluation, Trials and Studies Coordinating Centre.

**Contributors**

FMW and JE developed the initial idea for the trial, and all authors (except MP) have assisted with the development of the protocol, study design and refinement of study materials. PM and JE provided experience of primary care trials, and NB and PH provided clinical expertise. MR led the 2015 SSM Apps review. KM conducted the focus groups and interviews, and led trial preparations with RL and MP, and supported by trials and governance expertise from KW. CS is leading the statistical analysis. KM and FMW wrote the manuscript and revised it in response to critical revisions from all authors. FMW is the guarantor of the study.

**Funding**

This work was supported by FMW's Clinician Scientist award (RG 68235) from the National Institute for Health Research (NIHR). The views expressed in this publication are those of the authors and not necessarily those of the National Health Service, the NIHR or the Department of Health.

The paper also presents independent research funded/supported by the National Institute for Health Research (NIHR) Collaboration for Leadership in Applied Health Research & Care (CLAHRC) East of England, at Cambridgeshire and Peterborough NHS Foundation Trust. The views expressed are those of the author(s) and not necessarily those of the NHS, the NIHR or the Department of Health,

**Competing interests**

None declared.

**Ethics approval**

Cambridgeshire and Hertfordshire NHS REC committee, reference 16/EE/0248.

**Open Access**

This is an Open Access article distributed in accordance with the terms of the Creative Commons Attribution (CC BY 4.0) license, which permits others to distribute, remix, adapt and build upon this work, for commercial use, provided the original work is properly cited. See: http://creativecommons.org/licenses/by/4.0/



For peer review only - http://bmjopen.bmj.com/site/about/guidelines.xhtml

## REFERENCES


1. Cancer Research UK website. Available from: http://www.cancerresearchuk.org/health-professional/cancer-statistics/statistics-by-cancer-type/skin-cancer#heading-One (accessed 6th May 2017).
2. Arnold M, Holterhues C, Hollestein LM, et al. Trends in incidence and predictions of cutaneous melanoma across Europe up to 2015. J Eur Acad Dermatol Venereol. 2014; 28:1170-8.
3. Diepgen T, Mahler V. The epidemiology of skin cancer. British Journal of Dermatology. 2002;146(s61):1-6.
4. Balch CM, Gershenwald JE, Soong S-J, Thompson JF, Atkins MB, Byrd DR, et al. Final version of 2009 AJCC melanoma staging and classification. Journal of Clinical Oncology. 2009;27(36):6199-206.
5. Cancer Research UK website. Available from: http://www.cancerresearchuk.org/cancer-info/cancerstats/types/skin/survival/skin-cancer-survival-statistics#one (accessed 6th May 2017).
6. Sant M, Allemani C, Santaquilani M, Knijn A, Marchesi F, Capocaccia R, et al. EUROCARE-4. Survival of cancer patients diagnosed in 1995–1999. Results and commentary. European Journal of Cancer. 2009;45(6):931-91.
7. Abdel-Rahman M, Stockton D, Rachet B, Hakulinen T, Coleman M. What if cancer survival in Britain were the same as in Europe: how many deaths are avoidable? British Journal of Cancer. 2009;101:S115-S24.
8. Breitbart EW, Waldmann A, Nolte S, Capellaro M, Greinert R, Volkmer B, et al. Systematic skin cancer screening in Northern Germany. Journal of the American Academy of Dermatology. 2012;66(2):201-11.
9. Brunssen A, Waldmann A, Eisemann N, Katalinic A.Impact of skin cancer screening and secondary prevention campaigns on skin cancer incidence and mortality: A systematic review. J Am Acad Dermatol. 2017;76(1):129-139.e10.
10. Johnson MM, Leachman SA, Aspinwall LG, et al. Skin cancer screening: recommendations for data-driven screening guidelines and a review of the US Preventive Services Task Force controversy. Melanoma Manag. 2017;4(1),13-37.
11. Baughan P, O'Neill B, Fletcher E. Auditing the diagnosis of cancer in primary care: the experience in Scotland. British Journal of Cancer. 2009;101:S87-S91
12. Kebble S, Abel GA, Saunders CL, MacPhail S, Walter FM, Neal RD, et al. Variation in promptness of presentation among 10,297 patients subsequently diagnosed with one of 18 cancers: Evidence from a national audit of cancer diagnosis in primary care. Int J Cancer, 2014;135(5):1220-8.
13. Walter FM, Birt L, Cavers D. et al. 'This isn't what mine looked like': a qualitative study of symptom appraisal and help seeking in people recently diagnosed with melanoma. BMJ Open 2014; 4(7) e005566.
14. Cancer Research UK website. Available from: http://www.cancerresearchuk.org/health-professional/early-diagnosis-activities/be-clear-on-cancer/skin-cancer-campaign (accessed 6th May 2017).
15. Senior K. Smart phones: new clinical tools in oncology? The Lancet Oncology. 2011;12(5):429-30.




For peer review only - http://bmjopen.bmj.com/site/about/guidelines.xhtml

16. Korner A, Coroiu A, Martins C. et al. Predictors of skin self-examination before and after a melanoma diagnosis: the role of medical advice and patient's level of education. Int Arch Med 2013; 6(1):8.

17. Hamidi R, Peng D, Cockburn M. Efficacy of skin self-examination for the early detection of melanoma. Int J Dermatol. 2010; 49(2): 126-34.

18. Carli P, De Giorgi V,Palli D et al. Dermatologist detection and skin self-examination are associated with thinner melanomas: results from a survey of the Italian Multidisciplinary Group on Melanoma. Arch Dermatol. 2003; 139(5):607-12.

19. Korner A, Drapeau M, Thombs BD, et al. Barriers and facilitators of adherence to medical advice on skin self-examination during melanoma follow-up care. BMC Dermatol 2013; 13:3.

20. Murchie P, Allan JL, Brant W, Dennis M, Hall S, Masthoff J, et al. Total skin self-examination at home for people treated for cutaneous melanoma: development and pilot of a digital intervention. BMJ Open. 2015;5(8):e007993.

21. Craig P, Dieppe P, Macintyre S, Michie S, Nazareth I, Petticrew M. Developing and evaluating complex interventions: the new Medical Research Council guidance. BMJ 2008; 337: a1655–a1655

22. Usher-Smith JA, Kassianos A, Emery JD, Abel GA, Teoh Z, Hall S, Neal RD, Murchie P, Walter FM. Identifying people at higher risk of melanoma across the UK: a primary care based electronic survey. Brit J Dermatol. 2017 Apr;176(4):939-948.

23. Williams LH, Shors AR, Barlow WE, Solomon C, White E. Identifying Persons at Highest Risk of Melanoma Using Self-Assessed Risk Factors. J Clin Exp Dermatol Res 2011;2:pii:1000129, doi:10.4172/2155-9554.1000129.

24. Kassianos, A. P., Emery, J. D., Murchie, P., & Walter, F. M. (2015). Smartphone applications for melanoma detection by community, patient and generalist clinician users: a review. British Journal of Dermatology (6), 1507-1518.

25. Braun V, Clarke V. Using thematic analysis in psychology. Qual Res Psychol 2006;3:77–101.

26. Walter FM, Rubin G, Bankhead C, Morris HC, Hall N, Mills K, Dobson C, Rintoul RC, Hamilton W, Emery J. Symptoms and other factors associated with time to diagnosis and stage of lung cancer: a prospective cohort study. Brit J Cancer 2015; 112 Suppl 1:S6-13.

27. Walter FM, Emery JD, Mendonca S, Hall N, Morris HC, Mills K, Dobson C, Bankhead C, Johnson M, Abel GA, Rutter MD, Hamilton W, Rubin GP. Symptoms and patient factors associated with longer time to diagnosis for colorectal cancer: results from a prospective cohort study. Brit J Cancer 2016; 115(5): 533-541.

28. Walter FM, Mills K, Mendonca S, Abel G, Basu B, Carroll N, Hamilton W, Rubin GP, Emery JD. Pancreatic cancer: a prospective cohort study of symptoms and patient factors associated with diagnostic intervals and stage at diagnosis. Lancet Gastroenterology & Hepatology, October 4, 2016 http://dx.doi.org/10.1016/S2468-1253(16)30079-6

29. Glanz K, Gelle AC, Shigaki D, Maddock JE, Isnec MR. A randomized trial of skin cancer prevention in aquatics settings: the Pool Cool program. Health Psychol. 2002; 21(6):579-587.



For peer review only - http://bmjopen.bmj.com/site/about/guidelines.xhtml

30. Manne S, Lesin S. Prevalence and correlates of sun protection and skin self-examination practices among cutaneous malignant melanoma survivors. J Behav Med. 2006; 29(5):419-34.

31. Moye MS, King SM, Rice ZP, DeLong LK, Seidler AM, Veledar E, Curiel-Lewandrowski C, Chen SC. Effects of total-body digital photography on cancer worry in patients with atypical mole syndrome. JAMA Dermatol. 2015; 151(2):137-43.

32. Lerman C, Trock B, Rimer BK, Jepson C, Brody D, Boyce A. Psychological side effects of breast cancer screening. Health Psychol. 1991;10(4):259-267.

33. Smith S, Fielding S, Murchie P, Johnston M, Wyke S, Powell R, et al. Reducing the time before consulting with symptoms of lung cancer: a randomised controlled trial in primary care. Br J Gen Pract. 2013;63(606):47-54. Epub 2013/01/23.

34. Smith SM, Murchie P, Devereux G, Johnston M, Lee AJ, Macleod U, et al. Developing a complex intervention to reduce time to presentation with symptoms of lung cancer. Br J Gen Pract. 2012;62(602):e605-15. Epub 2012/09/06.

35. Zigmond AS, Snaith RP. The hospital anxiety and depression scale. ACTA Psychiatr Scand 1983; 67:361-70.

36. Ware, J, Kosinski M, Keller SD. A 12-Item Short-Form Health Survey: construction of scales and preliminary tests of reliability and validity. Med Care 1996; 34(3):220-233.

37. Lyratzopoulos G, Saunders CL, Abel GA, McPhail S, Neal RD, Wardle J, Rubin GP. The relative length of the patient and the primary care interval in patients with 28 common and rarer cancers. Br J Cancer 2015; 112 Suppl:S35-40.

For peer review only



For peer review only - http://bmjopen.bmj.com/site/about/guidelines.xhtml

**Figure 1: The availability and suitability of smartphone SSM Apps for the MelaTools SSM Trial**

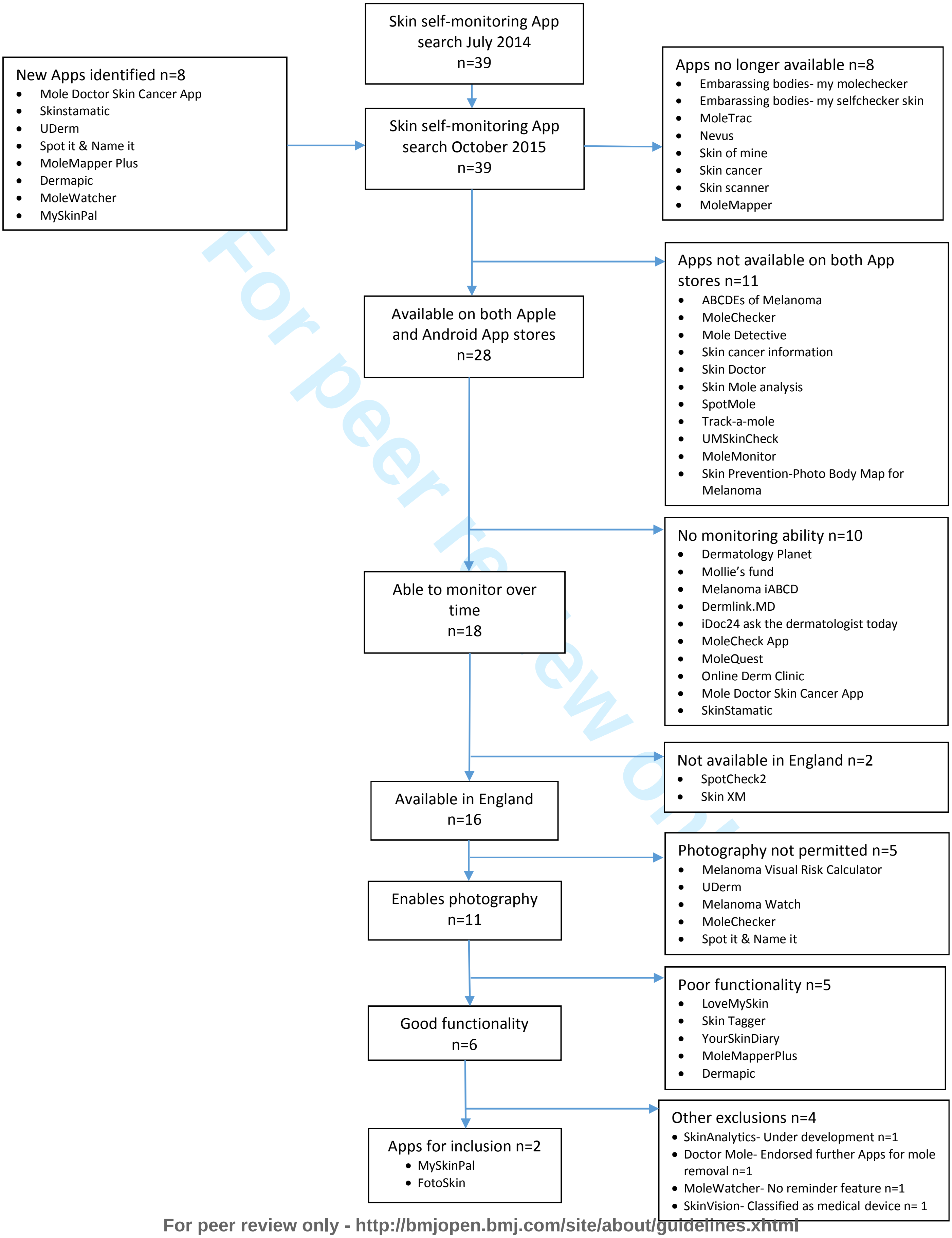


For peer review only

**Table 1: Piloting the intervention- consumer characteristics**

| | Participants (n=16) |
|---|---|
| ***Risk of Melanoma*** | |
| High risk of Melanoma (treated melanoma) | 8 |
| At above population risk | 5 |
| Unknown risk (relative of high risk participant) | 3 |
| ***Gender*** | |
| Male | 8 |
| Female | 8 |
| ***Age*** | |
| <35 years | 2 |
| 36-44 years | 1 |
| 45-54 years | 3 |
| 55-64 years | 7 |
| 65-74 years | 3 |
| ***Ethnicity*** | |
| White British | 15 |
| White Other | 1 |
| ***Education (highest qualification)*** | |
| GSCE or equivalent | 1 |
| A Level or equivalent | 3 |
| Vocational | 2 |
| Undergraduate degree | 3 |
| Postgraduate degree or professional qualification | 7 |
| ***Employment status*** | |
| Student | 1 |
| Work part-time | 5 |
| Work full-time | 4 |
| Homemaker | 1 |
| Retired | 5 |
| ***IMD Quintiles*** | |
| Least deprived 1 | 8 |
| 2 | 5 |
| 3 | 2 |
| 4 | 0 |
| Most deprived 5 | 0 |
| Unknown | 1 |

For peer review only



For peer review only - http://bmjopen.bmj.com/site/about/guidelines.xhtml

**Figure 2: MelaTools SSM Trial flowchart**

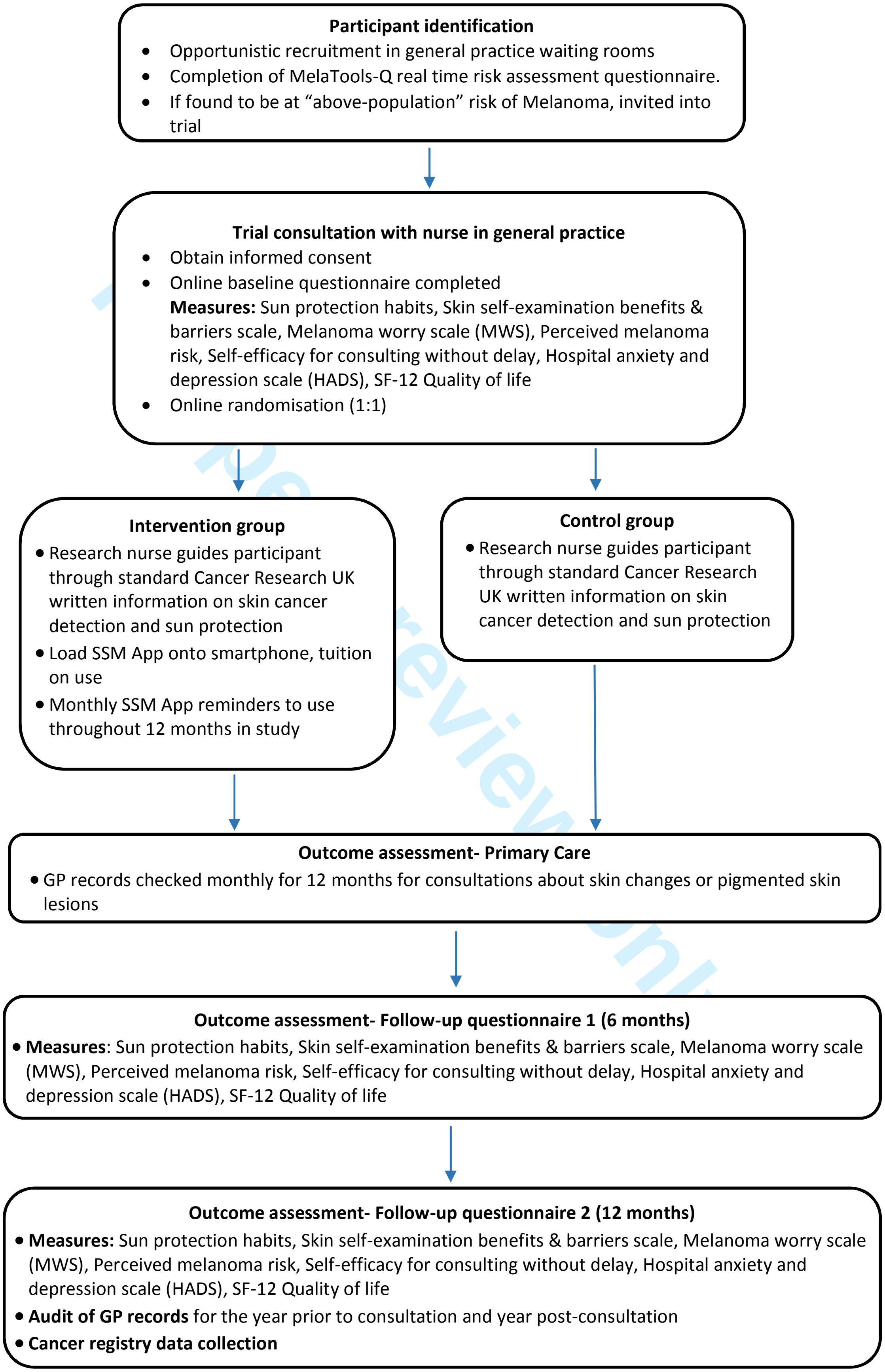




For peer review only - http://bmjopen.bmj.com/site/about/guidelines.xhtml

SPIRIT

STANDARD PROTOCOL ITEMS: RECOMMENDATIONS FOR INTERVENTIONAL TRIALS

SPIRIT 2013 Checklist: Recommended items to address in a clinical trial protocol and related documents*

| Section/item | Item No | Description | Addressed on page number |
|---|---|---|---|
| **Administrative information** | | | |
| Title | 1 | Descriptive title identifying the study design, population, interventions, and, if applicable, trial acronym | ________1______ |
| Trial registration | 2a | Trial identifier and registry name. If not yet registered, name of intended registry | ________2_____ |
| | 2b | All items from the World Health Organization Trial Registration Data Set | ______________ |
| Protocol version | 3 | Date and version identifier | ________1_____ |
| Funding | 4 | Sources and types of financial, material, and other support | ________11_____ |
| Roles and responsibilities | 5a | Names, affiliations, and roles of protocol contributors | _____1,11______ |
| | 5b | Name and contact information for the trial sponsor | ______________ |
| | 5c | Role of study sponsor and funders, if any, in study design; collection, management, analysis, and interpretation of data; writing of the report; and the decision to submit the report for publication, including whether they will have ultimate authority over any of these activities | ______________ |
| | 5d | Composition, roles, and responsibilities of the coordinating centre, steering committee, endpoint adjudication committee, data management team, and other individuals or groups overseeing the trial, if applicable (see Item 21a for data monitoring committee) | ______11_______ |



For peer review only - http://bmjopen.bmj.com/site/about/guidelines.xhtml

For peer review only

**Introduction**

| | | | |
|---|---|---|---|
| Background and rationale | 6a | Description of research question and justification for undertaking the trial, including summary of relevant studies (published and unpublished) examining benefits and harms for each intervention | ______3,4_____ |
| | 6b | Explanation for choice of comparators | ______________ |
| Objectives | 7 | Specific objectives or hypotheses | ______________ |
| Trial design | 8 | Description of trial design including type of trial (eg, parallel group, crossover, factorial, single group), allocation ratio, and framework (eg, superiority, equivalence, noninferiority, exploratory) | ______________ |

**Methods: Participants, interventions, and outcomes**

| | | | |
|---|---|---|---|
| Study setting | 9 | Description of study settings (eg, community clinic, academic hospital) and list of countries where data will be collected. Reference to where list of study sites can be obtained | ______6_______ |
| Eligibility criteria | 10 | Inclusion and exclusion criteria for participants. If applicable, eligibility criteria for study centres and individuals who will perform the interventions (eg, surgeons, psychotherapists) | _____6_______ |
| Interventions | 11a | Interventions for each group with sufficient detail to allow replication, including how and when they will be administered | ______7_______ |
| | 11b | Criteria for discontinuing or modifying allocated interventions for a given trial participant (eg, drug dose change in response to harms, participant request, or improving/worsening disease) | ______________ |
| | 11c | Strategies to improve adherence to intervention protocols, and any procedures for monitoring adherence (eg, drug tablet return, laboratory tests) | ______________ |
| | 11d | Relevant concomitant care and interventions that are permitted or prohibited during the trial | ______________ |
| Outcomes | 12 | Primary, secondary, and other outcomes, including the specific measurement variable (eg, systolic blood pressure), analysis metric (eg, change from baseline, final value, time to event), method of aggregation (eg, median, proportion), and time point for each outcome. Explanation of the clinical relevance of chosen efficacy and harm outcomes is strongly recommended | ________7_____ |
| Participant timeline | 13 | Time schedule of enrolment, interventions (including any run-ins and washouts), assessments, and visits for participants. A schematic diagram is highly recommended (see Figure) | ______17______ |



For peer review only - http://bmjopen.bmj.com/site/about/guidelines.xhtml

| | | | |
|---|---|---|---|
| Sample size | 14 | Estimated number of participants needed to achieve study objectives and how it was determined, including clinical and statistical assumptions supporting any sample size calculations | _____9_______ |
| Recruitment | 15 | Strategies for achieving adequate participant enrolment to reach target sample size | _____6______ |

**Methods: Assignment of interventions (for controlled trials)**

| | | | |
|---|---|---|---|
| Allocation: | | | |
| Sequence generation | 16a | Method of generating the allocation sequence (eg, computer-generated random numbers), and list of any factors for stratification. To reduce predictability of a random sequence, details of any planned restriction (eg, blocking) should be provided in a separate document that is unavailable to those who enrol participants or assign interventions | _____6_______ |
| Allocation concealment mechanism | 16b | Mechanism of implementing the allocation sequence (eg, central telephone; sequentially numbered, opaque, sealed envelopes), describing any steps to conceal the sequence until interventions are assigned | ______________ |
| Implementation | 16c | Who will generate the allocation sequence, who will enrol participants, and who will assign participants to interventions | ______________ |
| Blinding (masking) | 17a | Who will be blinded after assignment to interventions (eg, trial participants, care providers, outcome assessors, data analysts), and how | ______________ |
| | 17b | If blinded, circumstances under which unblinding is permissible, and procedure for revealing a participant's allocated intervention during the trial | ______________ |

**Methods: Data collection, management, and analysis**

| | | | |
|---|---|---|---|
| Data collection methods | 18a | Plans for assessment and collection of outcome, baseline, and other trial data, including any related processes to promote data quality (eg, duplicate measurements, training of assessors) and a description of study instruments (eg, questionnaires, laboratory tests) along with their reliability and validity, if known. Reference to where data collection forms can be found, if not in the protocol | ______6_____ |
| | 18b | Plans to promote participant retention and complete follow-up, including list of any outcome data to be collected for participants who discontinue or deviate from intervention protocols | ______________ |



For peer review only - http://bmjopen.bmj.com/site/about/guidelines.xhtml

| | | | |
|---|---|---|---|
| Data management | 19 | Plans for data entry, coding, security, and storage, including any related processes to promote data quality (eg, double data entry; range checks for data values). Reference to where details of data management procedures can be found, if not in the protocol | ______________ |
| Statistical methods | 20a | Statistical methods for analysing primary and secondary outcomes. Reference to where other details of the statistical analysis plan can be found, if not in the protocol | ___9_________ |
| | 20b | Methods for any additional analyses (eg, subgroup and adjusted analyses) | ______________ |
| | 20c | Definition of analysis population relating to protocol non-adherence (eg, as randomised analysis), and any statistical methods to handle missing data (eg, multiple imputation) | ______________ |
| **Methods: Monitoring** | | | |
| Data monitoring | 21a | Composition of data monitoring committee (DMC); summary of its role and reporting structure; statement of whether it is independent from the sponsor and competing interests; and reference to where further details about its charter can be found, if not in the protocol. Alternatively, an explanation of why a DMC is not needed | ___10_______ |
| | 21b | Description of any interim analyses and stopping guidelines, including who will have access to these interim results and make the final decision to terminate the trial | ______________ |
| Harms | 22 | Plans for collecting, assessing, reporting, and managing solicited and spontaneously reported adverse events and other unintended effects of trial interventions or trial conduct | ______________ |
| Auditing | 23 | Frequency and procedures for auditing trial conduct, if any, and whether the process will be independent from investigators and the sponsor | ______________ |
| **Ethics and dissemination** | | | |
| Research ethics approval | 24 | Plans for seeking research ethics committee/institutional review board (REC/IRB) approval | __11________ |
| Protocol amendments | 25 | Plans for communicating important protocol modifications (eg, changes to eligibility criteria, outcomes, analyses) to relevant parties (eg, investigators, REC/IRBs, trial participants, trial registries, journals, regulators) | ______________ |



For peer review only - http://bmjopen.bmj.com/site/about/guidelines.xhtml

| | | | |
|---|---|---|---|
| Consent or assent | 26a | Who will obtain informed consent or assent from potential trial participants or authorised surrogates, and how (see Item 32) | 6 |
| | 26b | Additional consent provisions for collection and use of participant data and biological specimens in ancillary studies, if applicable | |
| Confidentiality | 27 | How personal information about potential and enrolled participants will be collected, shared, and maintained in order to protect confidentiality before, during, and after the trial | 8 |
| Declaration of interests | 28 | Financial and other competing interests for principal investigators for the overall trial and each study site | 11 |
| Access to data | 29 | Statement of who will have access to the final trial dataset, and disclosure of contractual agreements that limit such access for investigators | |
| Ancillary and post-trial care | 30 | Provisions, if any, for ancillary and post-trial care, and for compensation to those who suffer harm from trial participation | |
| Dissemination policy | 31a | Plans for investigators and sponsor to communicate trial results to participants, healthcare professionals, the public, and other relevant groups (eg, via publication, reporting in results databases, or other data sharing arrangements), including any publication restrictions | |
| | 31b | Authorship eligibility guidelines and any intended use of professional writers | |
| | 31c | Plans, if any, for granting public access to the full protocol, participant-level dataset, and statistical code | |
| **Appendices** | | | |
| Informed consent materials | 32 | Model consent form and other related documentation given to participants and authorised surrogates | |
| Biological specimens | 33 | Plans for collection, laboratory evaluation, and storage of biological specimens for genetic or molecular analysis in the current trial and for future use in ancillary studies, if applicable | |

*It is strongly recommended that this checklist be read in conjunction with the SPIRIT 2013 Explanation & Elaboration for important clarification on the items. Amendments to the protocol should be tracked and dated. The SPIRIT checklist is copyrighted by the SPIRIT Group under the Creative Commons "Attribution-NonCommercial-NoDerivs 3.0 Unported" license.



For peer review only - http://bmjopen.bmj.com/site/about/guidelines.xhtml

# Protocol for the MelaTools Skin Self-Monitoring Trial: a phase II randomised controlled trial of an intervention for primary care patients at higher risk of melanoma

| | |
|---|---|
| Journal: | *BMJ Open* |
| Manuscript ID | bmjopen-2017-017934.R1 |
| Article Type: | Protocol |
| Date Submitted by the Author: | 15-Aug-2017 |
| Complete List of Authors: | Mills, Katie; University of Cambridge, Public Health & Primary Care<br>Emery, Jon; University of Melbourne, General Practice and Primary Care Academic Centre<br>Lantaff, Rebecca; University of Cambridge, Public Health & Primary Care<br>Radford, Michael; University of Cambridge, Public Health & Primary Care<br>Pannebakker, Merel; University of Cambridge, Public Health & Primary Care<br>Hall, Per; Cambridge University Hospitals NHS Foundation Trust<br>Burrows, Nigel; Cambridge University Hospitals NHS Foundation Trust<br>Williams, Kate; University of Cambridge, Public Health & Primary Care<br>Saunders, Catherine; University of Cambridge, Cambridge Centre for Health Services Research<br>Murchie, Peter; University of Aberdeen, Division of Applied Health Science<br>Walter, Fiona; University of Cambridge, Dept of Public Health and Primary Care |
| <b>Primary Subject Heading</b>: | General practice / Family practice |
| Secondary Subject Heading: | Dermatology |
| Keywords: | melanoma, skin cancer, randomised controlled trial, risk-stratified, PRIMARY CARE |
| | |

# Protocol for the MelaTools Skin Self-Monitoring Trial: a phase II randomised controlled trial of an intervention for primary care patients at higher risk of melanoma

Mills K[1], Emery JD[2,1], Lantaff R[1], Radford M[3], Pannebakker MM[1], Hall P[4], Burrows NP[4], Williams KM[1], Saunders CL[1], Murchie P[5], Walter FM[1,2]

[1] The Primary Care Unit, Department of Public Health and Primary Care, University of Cambridge, CB1 8SR, UK
[2] Department of General Practice and the Centre for Cancer Research, Faculty of Medicine, Dentistry and Health Science, Victorian Comprehensive Cancer Centre, University of Melbourne, Australia
[3] Tennyson House Surgery, Chelmsford CM1 4HW, UK
[4] Cambridge University Hospitals NHS Foundation Trust, Hills Road, Cambridge CB2 0QQ, UK
[5] Division of Applied Health Science, Centre of Academic Primary Care, University of Aberdeen, Polwarth Building, Foresterhill, Aberdeen AB25 2ZD, UK

**Correspondence to:** Katie Mills ko298@medschl.cam.ac.uk

**Key words:** melanoma, skin cancer, randomised controlled trial, risk-stratified, primary care

**Word Count**: 3876
2 figures, 1 table

For peer review only



For peer review only - http://bmjopen.bmj.com/site/about/guidelines.xhtml

## ABSTRACT

### Introduction

Melanoma is the fifth commonest cancer in the UK. Incidence rates have quadrupled over the last 30 years and continue to rise, especially among younger people. As routine screening of the general population is not currently recommended in the UK, a focus on secondary prevention through early detection and prompt treatment in individuals at increased risk of melanoma could make an important contribution to improve melanoma outcomes. This paper describes the protocol for a phase II, multisite, randomised controlled trial, in the primary care setting, for patients at increased risk of melanoma. A skin self-monitoring smartphone 'App' was used to improve symptom appraisal and encourage help-seeking in primary care, thereby promoting early presentation with skin changes suspicious of melanoma.

### Methods and analysis

We aim to recruit 200 participants from general practice waiting rooms in the East of England. Eligible patients are those identified at higher melanoma risk (using a real-time risk assessment tool), without a personal history of melanoma, aged 18 to 75 years. Participants will be invited to a primary care nurse consultation, and randomised to the Intervention group (standard written advice on skin cancer detection and sun protection, loading of a skin self-monitoring 'App' onto the participant's smartphone, and instructions on use including self-monitoring reminders) or Control group (standard written advice alone). The primary outcomes are consultation rates for changes to a pigmented skin lesion, and the patient interval (time from first noticing a skin change to consultation). Secondary outcomes include patient sun-protection behaviours, psychosocial outcomes, and measures of trial feasibility and acceptability.

### Ethics and dissemination

NHS ethical approval has been obtained from Cambridgeshire and Hertfordshire research ethics committee (REC reference 16/EE/0248). The findings from the MelaTools SSM Trial will be disseminated widely through peer-reviewed publications and scientific conferences.

**Trial registration number:** ISCTRN registry, ISCTRN16061621


For peer review only



For peer review only - http://bmjopen.bmj.com/site/about/guidelines.xhtml

**Strengths and limitations of this study**

- The MelaTools SSM Trial is among the first, in the UK or internationally, to risk stratify the primary care population in order to target an intervention to improve timeliness of melanoma detection.
- It uses digital technology to encourage people at higher risk of melanoma to self-monitor their skin and present to their physician with any concerns.
- The trial aims to establish the feasibility and acceptability of undertaking skin self-monitoring among people at higher risk of melanoma; it will also report on consultation rates and includes 6- and 12-month participant follow-up.
- As this is a feasibility trial the main limitation is that the small sample size will limit meaningful interpretation of the clinical outcomes; furthermore, the sample may not be fully representative of the UK adult population as it excludes people who do not own a smartphone, and those with physical disorders severe enough to hamper smartphone use.
- Information from this trial will enable planning of a larger phase III trial to further assess the impact of the use of a smartphone App to encourage skin self-monitoring on clinical outcomes for melanoma.

For peer review only



For peer review only - http://bmjopen.bmj.com/site/about/guidelines.xhtml

## INTRODUCTION

Malignant melanoma is the leading cause of skin cancer deaths in the UK with 2,459 in 2014 (1). Melanoma skin cancer incidence has quadrupled over the last 30 years, and continues to rise (2). Although melanoma is more common with increasing age, around half of melanoma in the UK each year are diagnosed in people aged less than 65 (3, 4). Risk factors include fair skin, family history of melanoma, multiple naevi, and sun damage. Melanoma is associated with significant morbidity, and the thickness of the lesion at diagnosis is the most important prognostic factor: stage 1 disease has 5-year survival rates of over 95%, compared to less than 25% for stage 4 disease (5). The UK has lower 1- and 5-year melanoma survival rates than comparable countries in Europe (6). Diagnostic delays are thought to contribute to this, and there is evidence of avoidable delay (7). Evidence from the SCREEN project in Germany, conducted between 2003 and 2004, suggested that population screening may have an impact on melanoma incidence and 5-year mortality (8, 9). However, this evidence is controversial, and routine screening of the general population is not currently recommended anywhere worldwide, although some countries (Australia, New Zealand, Germany, the Netherlands and the UK) recommend regular skin checks and/or self-examination for certain subsets of patients at increased risk of melanoma (10). Therefore, a focus on targeted population screening could make an important contribution to melanoma outcomes through early, timely detection and prompt treatment.

Patient pathways to presentation and management in primary care are key determinants in cancer outcomes. When compared with people diagnosed with other cancers, those diagnosed with melanoma have the second longest median time between first noticing a symptom and presenting to primary care (patient interval) (11, 12). The patient interval could potentially be reduced by providing patients with clear information on the signs and symptoms of melanoma and guidance on monitoring skin changes (13), through community campaigns such as the Be Clear on Cancer skin cancer campaign (14). More targeted approaches could focus on individuals at higher risk.

Smartphones offer considerable potential to promote earlier presentation by people with cancer, including melanoma (15). More than 70% of the UK adult population now own a smartphone (Techtracker), and there are many smartphone applications ('Apps') focusing on health issues, presenting new opportunities for risk assessment, symptom appraisal, monitoring symptoms and signs over time, and cues to seek professional advice. Furthermore, up to 75% of melanomas are detected by people or their family/friends, rather than health care professionals. To maximise the effectiveness of skin self-monitoring (SSM) the person performing the examination should be able to identify skin changes and features of skin lesions which could indicate melanoma, yet recent studies have demonstrated that suspicious signs of skin cancer may not be widely known (16,13). Important international evidence has shown that it is possible to educate patients and the public: newly diagnosed Italian melanoma patients who performed self-skin examination were found to have thinner tumours (17), and Australian melanoma patients were found to adhere to medical advice on skin self-examination during follow-up care (18). A recent Scottish study has demonstrated melanoma patients are prepared to use digital technology



For peer review only - http://bmjopen.bmj.com/site/about/guidelines.xhtml

to support them in conducting SSM during follow-up (19). We therefore set out to explore using mobile technology for SSM among people at higher risk of melanoma in the primary care setting to encourage timely consultation for possible melanoma.

**Preliminary Phase I research**

Guided by the Medical Research Council (MRC) framework on developing and evaluating complex interventions (20), three Phase I studies were conducted to: (i) assess the feasibility and acceptability of defining a population at higher risk of melanoma from UK general practice; (ii) review the availability of suitable smartphone SSM Apps, and (iii) pilot the intervention.

***(i) Assessing the feasibility and acceptability of defining a population at higher risk of melanoma from UK general practice***

The MelaTools-Q Study findings have already been reported (21). Briefly, 7,742 patients (86% of those approached) were recruited from the waiting rooms of 22 general practices in three UK regions: Eastern England, Northeast Scotland and North Wales. Using tablet computers, they completed an electronic questionnaire incorporating the Williams melanoma risk prediction model (22), including items relating to 7 risk factors: gender, age, natural hair colour at age 15, number of severe sunburns aged 2-18, prior non-melanoma skin cancer, number of raised moles on both arms, and density of freckles on both arms before age 20. The study showed that, after weighting to the age and sex distribution, the lower and upper quartile cut-offs used by Williams *et al.*, 25 and 34, would allow between 4% and 20% of the population to be identified as higher risk, and those groups would contain 30% and 60%, respectively of individuals who would develop melanoma. Therefore, real-time risk assessment for melanoma in UK primary care is both feasible and acceptable.

***(ii) Reviewing the availability of suitable smartphone SSM Apps***

Our initial review, conducted in July 2014 identified 39 smartphone Apps available from Apple and Android App online stores (23). However, when the same search strategy was re-run in September 2015, 8 new SSM Apps were identified, while 8 of the previously identified SSM Apps were no longer available.  Among the 39 newly-identified SSM Apps, 11 were not available in both Apple and Android App stores; 10 did not include a monitoring function; 5 did not include photography of skin lesions; 5 had poor functionality; 2 were not available in England; 2 were classified as medical devices; one was under development, and one had no reminder feature, deemed vital for monitoring functionality. Two SSM Apps were considered potentially most suitable to include in the intervention (see Figure 1).

***(iii) Piloting the intervention***

Qualitative research, using focus groups and interviews, was conducted with individuals at increased risk of melanoma to provide in-depth understanding of consumer views on the usefulness and usability of the two selected SSM Apps. Eight people at increased risk of melanoma were recruited for two focus groups from a local melanoma patient support group; three of their relatives also took part.  People were also recruited in the waiting rooms of two suburban general practices and completed the Melatools-Q risk assessment tool (21). Five identified to be at increased risk of melanoma were invited to participate in two other focus groups. A



For peer review only - http://bmjopen.bmj.com/site/about/guidelines.xhtml

total of 16 participants tested at least one of the SSM Apps. The participant characteristics are shown in Table 1.

The four focus groups were conducted by an experienced qualitative researcher (KM) and research assistant (RL), either at the recruiting general practice or university premises; each lasted approximately 90 minutes, was audio-recorded, and the recordings transcribed verbatim. Each participant was initially helped to load the two SSM Apps on their smartphones, and given time to familiarise themselves with their use. Thematic analysis (24) showed that, overall, participants in all four groups and at all strata of increased risk of melanoma felt that using a SSM App would be helpful and achievable. Many felt that using a SSM App would enhance their current skin self-monitoring habits: *"I look at the moles and I just keep an eye on them" (Male, above-population risk, group 3).* Many also felt that using a SSM App could help reduce their worry about developing skin cancer: *"cos my dad had cancer recently so [my mother] says we're at a higher risk, so she goes on about that" (Female, above-population risk, group 4).* Some felt that using a SSM App would guide their help-seeking behaviour for skin changes or moles: *"It's that knowledge, if it's, you know, three months, whatever it is, if you can actually say look there is a visible change then you're getting a bit of information that they probably could do with…you've got physical evidence, and then the confidence to go to…" (Male, above-population risk, group 4).* Several also commented on additional features on smartphones which might help prevent development of future melanomas: *"I've got a UV index on my phone so I can always check what that is, so putting sun cream on." (Male, high risk, group 2).*

The five participants recruited via primary care were invited to complete a diary one and two months later, and then participate in a telephone interview after the third month. Diary records and thematic analysis of the interviews (as above) confirmed that participants used many features of both SSM Apps, including: taking photographs, mapping these to body parts, comparing photographs over time, and using the analysis function (available in only one of the Apps). There was unanimous agreement between participants about the App which was easier to use (MySkinPal), and most reported that they would continue to use it after the study, particularly as they found the App's notifications the most efficient method to prompt them to complete future skin self-monitoring. Despite this, several also reported that they found taking photographs quite challenging, and that they had to recruit a family member to assist them in taking photographs to compare skin changes over time. Furthermore, while regularly self-monitoring their skin during the study, most participants reported worrying about skin cancer 'sometimes', seeking further information about skin changes or moles via the internet, speaking to friends and family, and visiting their general practice.

In conclusion, this preliminary Phase I work enabled consumer choice of the more user-friendly SSM App, resulted in modifications to the language used in the trial consultation, and led to the development of the MelaTools SSM Trial. The trial aims to assess the effect of using a SSM App compared with standard information about detecting skin cancer on consultation rates and the patient interval (the time from first noticing a skin change/mole to consultation with a primary care healthcare



For peer review only - http://bmjopen.bmj.com/site/about/guidelines.xhtml

professional) among patients at increased risk of melanoma, identified via primary care. In addition, to obtain preliminary estimates of effect across a range of outcome measures to inform selection of the primary outcome for a definitive phase III trial.

## METHODS AND ANALYSIS

### Study design and setting

The phase II trial is a multi-site randomised controlled trial (Figure 2), set in 11 general practices across Eastern England. Those who meet the eligibility criteria and consent to participate are randomised 1:1 into either the control or intervention group. Randomisation is being performed using an online system provided by the Clinical Trials Unit based at Kings College London.

### Study participants

#### *Eligibility criteria*

Eligible participants are individuals aged between 18 and 75 years who own a smartphone (Apple or Android), and, on completion of the Melatools-Q risk assessment tool (21), are found to be at increased risk of melanoma. Participants are able to read and write English and to give informed consent.

#### *Exclusion criteria*

Exclusion criteria are previous diagnosis of melanoma, severe psychiatric or cognitive disorders, or a physical disorder severe enough to inhibit the use of a smartphone.

#### *Participant recruitment*

Potentially eligible individuals are approached by research nurses or research assistants in GP waiting areas at different times of the day and different days of the week, in order to ensure that a broad range of ages, gender, and educational level are approached. Those meeting initial screening criteria are given a tablet computer that includes a short version of the participant information sheet and an online consent form agreeing to have their risk assessed and be considered for the trial. They complete the MelaTools Q study risk assessment tool (21) which stratifies respondents into 'population risk' (majority) and 'increased risk', using a cut-off score in the Williams model of 25. People at 'population risk' are thanked and given the Cancer Research UK leaflets on risk factors of melanoma and melanoma detection advice. People at increased risk are invited to participate in the trial, and given the full-length trial Participant Information Sheet and an appointment time within 14 days for the primary care nurse trial consultation. Individuals not wishing to take part are asked their reason for declining.

#### *Control and Intervention groups and randomisation*

Informed written consent is obtained from all participants at the start of the trial consultation (supplementary file 1). After completion of a baseline questionnaire, the trial nurse guides the participant through the Cancer Research UK leaflets on "Be SunSmart cut your cancer risk" and "Skin cancer - How to spot the signs and symptoms". Online randomisation is then performed: those randomised to the control group are thanked for their participation and both groups are given the Cancer Research UK leaflets to take home.



For peer review only - http://bmjopen.bmj.com/site/about/guidelines.xhtml

***Intervention group***
Participants randomised to the intervention group are assisted with loading the skin self-monitoring App onto their smartphone by the trial nurse, who continues by giving instructions on its use, supported by written instruction sheets. The core functions of the App, MySkinPal, include: full body scan, body diagrams, photo source and browser, information on the signs of melanoma, regular reminders, ability to select moles to share with a doctor, and motivation to use including awards/achievements. The nurse ensures the App's monthly reminder notification is switched on to prompt each participant regularly. Finally, each intervention participant is given an Apple Apps store/Google Play voucher (up to £5) to pay for the App.

**Outcomes and measures**
The primary outcomes of this trial are *consultation rates* for pigmented skin lesions, and the *patient interval. Consultation rates* for changes to an existing pigmented skin lesion or concerns about a new pigmented skin lesion for the 12 months prior to the trial and the 12 months after the trial consultation will be collected via searching general practice electronic medical records using a clinical notes audit for every participant. The *patient interval* is the time from first noticing a skin change to first consultation. These data will be collected monthly by searches of general practice electronic medical records for all skin changes or pigmented skin lesions presented to their GP or practice nurse during the 12 months following the trial consultation. When a participant consults about a pigmented skin lesion, they receive a Skin Questionnaire to complete. This skin questionnaire uses the Symptom Study instrument modified for melanoma symptoms; a participant-completed questionnaire collecting data on symptoms and their duration prior to consultation, (24-27).

Secondary measures and outcomes (measured at baseline, 6- and 12-months) include:

(1) *Demographics and clinical variables:* age, gender, marital status, postcode, highest education level, occupation, past history of skin cancer (melanoma, Squamous Cell Carcinoma, Basal Cell Carcinoma), skin and hair type (density of freckles on arms before age 20, natural hair colour at age 15, number of severe sunburns aged 2-18), number of raised moles on both arms, measured at baseline only (21,22), collected as part of the baseline eligibility assessment;

(2) *Sun protection habits scale*: developed by Glanz et al in the US for a multicomponent skin cancer prevention programme, this comprises 5 items measured using a 4 point Likert scale, and relating to use of sun protection, sun and sunbed habits, and episodes of sunburn in the previous year (28), as we hypothesise that the App reminders will not only prompt people but also reinforce the messages on sun protection;

(3) *Skin Self-Examination benefits and barriers scale*: validated by Manne and Lessin in the US among melanoma survivors, and developed from previous work on mammography and family members of patients with colorectal



For peer review only - http://bmjopen.bmj.com/site/about/guidelines.xhtml

cancer (29). The benefits scale has seven items (α=.71) and the barriers scale has ten items (α=.74);

(4) *Melanoma Worry Scale (MWS):* validated by Moye et al in the US (30), an adapted from the Breast Cancer Worry Scale (31), this measure comprises four items, scored 1 to 4, with possible scores ranging from 4 to 17, and higher scores indicating higher levels of worry;

(5) *Perceived Melanoma Risk*: drawn from Manne and Lessin's measures (29), these two items have been widely used for melanoma and other cancer risk assessments to assess estimated percent risk of developing melanoma, and perceived risk compared with a person of the same age (relative risk);

(6) *Self-Efficacy for consulting without delay*: A 10-item self-completed scale summed to score 10-100, was used in a primary care trial for lung symptoms, and showed good internal reliability (Cronbach α =0.85) (32, 33). It has been adapted for this trial, and reduced to 8 items, e.g. 'How confident are you that you can make an appointment to see a doctor when…… you can't get an appointment with your usual doctor?';

(7) *Hospital anxiety and depression scale (HADS):* This 14-item self-completed scale has been widely used to measure distress and has been extensively validated and shown to perform well in a wide range of populations (mean Cronbach α = 0.82; sensitivity and specificity 0.80) (34);

(8) *SF-12 Quality of life scale*: a 12-item version of the SF-36 that is widely used and validated to measure functional health and well-being (35), e.g. 'During the past 4 weeks, have you had any of the following problems with your work or other regular daily activities as a result of your physical health?.....'

Other outcome measures include:

(9) *Trial feasibility and acceptability*, including: data on patient recruitment and attrition rates, reasons for attrition, response rates to Skin Symptom Questionnaires for measuring the patient interval to inform decisions about a future phase III trial.

(10) *Melanoma incidence across participating practices*, to contextualise trial findings and after 5 years. This will be identified through GP electronic medical records and the National Cancer Registration Service.

**Measurement timings and study end points**

At the trial consultation all participants will be fully informed about trial follow-up procedures. The participant-reported measures (numbers 1-8 above) are completed in person at baseline, and online at follow-up 1 (6 months) and follow-up 2 (12 months), with the exception of the skin symptom questionnaire as already described, which is sent to participants by postal mail within a month of any consultation for skin changes or pigmented skin lesions.

After trial completion, a final audit of the GP electronic medical records will be run to identify all skin consultations for the 12 months during the trial as well as the previous 12 months. Melanoma incidence findings will also be collected at the end of the 12 month follow up period and at 5 years. These data will not be collected for participants who formally withdraw from the trial.



For peer review only - http://bmjopen.bmj.com/site/about/guidelines.xhtml

**Participant data and study management**

All participants are allocated a unique identifying code. Melanoma risk assessment data, baseline questionnaire, and follow up questionnaires data are stored on a custom built database on a secure server hosted by the Outcome Registry Intervention and Operation Network (ORION), Clinical Neurosciences department at the University of Cambridge.

**Sample size consideration**

Based on our recent MelaTools Q study with an identical screening step (response rate of 86%), we anticipate that we will need to approach approximately 2,000 people from 10 general practice waiting rooms for about 1,600 to complete the Melatools Q risk assessment tool (23). About 25%, 400 people, will be identified as increased risk and be eligible to participate in the trial. Based on previous research we would expect approximately 50% of these to attend their trial consultation and undergo randomisation in order to reach our target of 200 participants.

We will use information on recruitment, attrition, and data completeness, and preliminary estimates of effect sizes where appropriate, to inform the sample size calculation for a future phase III trial.

**Statistical analysis**

All randomised patients will be considered eligible for inclusion in the analysis in accordance with the intention-to-treat analysis principle. As this is a phase II trial, fully describing and characterising the extent and nature of the missing data is an important part of the analysis. For the outcome analysis, appropriate methods for dealing with missing endpoint data will be detailed in the statistical analysis plan and be informed by a blinded review of the data. The baseline characteristics of the two arms will be described using summary statistics. Possible consent bias will be assessed by comparing demographic and clinical variables of participants against those who declined participation, and possible differential attrition will be assessed by comparing baseline characteristics of those who withdraw or die against those who remain in the trial. These comparisons will be performed using a two sample t-test (or non-parametric equivalent) for continuous variables and chi-square test for categorical variables.

The primary analysis will be a comparison between the two groups on the consultation rate for skin changes/pigmented skin lesion using a Poisson regression model. Comparisons between groups on binary secondary endpoints will be performed using logistic regression. Comparisons between groups on continuous secondary endpoints will be undertaken using a linear model that includes the baseline value where applicable. A linear model may not be appropriate for the secondary outcome Patient Interval, as this is expected to be right skewed (36). We will explore the nature and extent of the skewness and analyse Patient Interval using a linear model if possible, adopting other approaches as appropriate, including transformation or categorisation of the Patient Interval, or methods for statistical inference based on bootstrap resampling. The analyses performed on the primary and secondary endpoints will be repeated adjusting for additional baseline covariates as part of a sensitivity analysis. Point estimates of the intervention effect



For peer review only - http://bmjopen.bmj.com/site/about/guidelines.xhtml

will be presented with 95% confidence intervals and two-sided p-values. Unadjusted p-values from secondary analyses will be interpreted in proper context and be clearly labelled.

## ETHICS AND DISSEMINATION

### Ethical approval

This trial is sponsored by the School of Clinical Medicine at the University of Cambridge. Ethical approval for the MelaTools SSM trial was granted on 11th July 2016 by the Cambridgeshire and Hertfordshire NRES research ethics committee (reference number 16/EE/0248). Health Research Authority approval (HRA) was granted on 22nd July 2016. R&D approvals have been obtained from the relevant Trusts. All substantial amendments are approved by the NHS Research Ethics committee responsible for the trial, in addition to approval by HRA and NHS R&D. Investigators are kept up to date with relevant changes via regular management group meetings.

### Data monitoring

Due to the non-medicinal and low-risk nature of the trial a data monitoring committee will not be needed. The trial steering committee (the CI, collaborators and researchers, statistician and PPIE representatives) will meet 6 monthly from the start of the trial and will monitor trial progress, approve a data analysis plan, and will ensure the trial runs in accordance with the protocol and applicable standard operating procedures. The CI will take responsibility for data monitoring and ethics, and will be responsible for communicating important protocol modifications to relevant parties.

### Expected findings, implications and dissemination

Twelve-month follow-up will complete in January 2018. We expect that the trial will prove to be both feasible and acceptable to participants without causing them significant anxiety or harm. We do not expect to be able to draw any significant clinical conclusions from this small trial, therefore we expect to be use these findings to support a funding application for a larger Phase III trial aiming to be sufficiently powered to enable evaluation of clinical outcomes. We are also interested in further work exploring the usefulness of different apps for different groups of consumers.

We will submit the findings of our research for publication in highly cited and open access peer-reviewed journals, and the findings will also be presented at national and international conferences. We will also provide a summary of our findings on the MelaTools programme website MelaTools.org.



For peer review only - http://bmjopen.bmj.com/site/about/guidelines.xhtml

For peer review only

**Acknowledgements**
The authors are grateful to the patients and general practices who contributed to the pilot study. The authors would also like to thank the MelaTools Steering Committee for their scientific support and comments throughout the design and set-up of the trial: Pippa Corrie, Katharine Acland, Juliet Usher-Smith, Ed Wilson, Margaret Johnson, Simon Rodwell and Patricia Fairbrother. This study is supported by the UK Clinical Research Collaboration-registered King's Clinical Trials Unit at King's Health Partners, which is part-funded by the NIHR Biomedical Research Centre for Mental Health at South London and Maudsley NHS Foundation Trust and King's College London and the NIHR Evaluation, Trials and Studies Coordinating Centre.

**Contributors**
FMW and JE developed the initial idea for the trial, and all authors (except MP) have assisted with the development of the protocol, study design and refinement of study materials. PM and JE provided experience of primary care trials, and NB and PH provided clinical expertise. MR led the 2015 SSM Apps review. KM conducted the focus groups and interviews, and led trial preparations with RL and MP, and supported by trials and governance expertise from KW. CS is leading the statistical analysis. KM and FMW wrote the manuscript and revised it in response to critical revisions from all authors. FMW is the guarantor of the study.
The International Committee of Medical Journal Editors (ICMJE) authorship eligibility guidelines have been followed and no professional writers have been used.

**Funding**
This work was supported by FMW's Clinician Scientist award (RG 68235) from the National Institute for Health Research (NIHR). The views expressed in this publication are those of the authors and not necessarily those of the National Health Service, the NIHR or the Department of Health. The funding source has no role in the design of this study, the interpretation of data, or the decision to submit results.
The paper also presents independent research funded/supported by the National Institute for Health Research (NIHR) Collaboration for Leadership in Applied Health Research & Care (CLAHRC) East of England, at Cambridgeshire and Peterborough NHS Foundation Trust. The views expressed are those of the author(s) and not necessarily those of the NHS, the NIHR or the Department of Health,

**Competing interests**
None declared.

**Ethics approval**
Cambridgeshire and Hertfordshire NHS REC committee, reference 16/EE/0248, protocol version 2 24th June 2016. Requests for the final dataset should be addressed to the corresponding author.

**Trial progress**
The study was registered in the ISRCTN Trials Registry on 17th August 2016, the first participant was recruited on 22nd August 2016, enrolment was completed on 6th January 2017, and the participants continue to be followed up in the study, with an estimated finish date of end January 2018.



For peer review only - http://bmjopen.bmj.com/site/about/guidelines.xhtml

**Open Access**
This is an Open Access article distributed in accordance with the terms of the Creative Commons Attribution (CC BY 4.0) license, which permits others to distribute, remix, adapt and build upon this work, for commercial use, provided the original work is properly cited. See: http://creativecommons.org/licenses/by/4.0/

**Figure Legends**
Figure 1: The availability and suitability of smartphone SSM Apps for the MelaTools SSM Trial
Figure 2: MelaTools SSM Trial flowchart

## REFERENCES

1. Cancer Research UK website. Available from: http://www.cancerresearchuk.org/health-professional/cancer-statistics/statistics-by-cancer-type/skin-cancer#heading-One (accessed 6th May 2017).

2. Arnold M, Holterhues C, Hollestein LM, et al. Trends in incidence and predictions of cutaneous melanoma across Europe up to 2015. J Eur Acad Dermatol Venereol. 2014; 28:1170-8.

3. Diepgen T, Mahler V. The epidemiology of skin cancer. British Journal of Dermatology. 2002;146(s61):1-6.

4. Balch CM, Gershenwald JE, Soong S-J, Thompson JF, Atkins MB, Byrd DR, et al. Final version of 2009 AJCC melanoma staging and classification. Journal of Clinical Oncology. 2009;27(36):6199-206.

5. Cancer Research UK website. Available from: http://www.cancerresearchuk.org/cancer-info/cancerstats/types/skin/survival/skin-cancer-survival-statistics#one (accessed 6th May 2017).

6. Sant M, Allemani C, Santaquilani M, Knijn A, Marchesi F, Capocaccia R, et al. EUROCARE-4. Survival of cancer patients diagnosed in 1995–1999. Results and commentary. European Journal of Cancer. 2009;45(6):931-91.

7. Abdel-Rahman M, Stockton D, Rachet B, Hakulinen T, Coleman M. What if cancer survival in Britain were the same as in Europe: how many deaths are avoidable? British Journal of Cancer. 2009;101:S115-S24.

8. Breitbart EW, Waldmann A, Nolte S, Capellaro M, Greinert R, Volkmer B, et al. Systematic skin cancer screening in Northern Germany. Journal of the American Academy of Dermatology. 2012;66(2):201-11.

9. Brunssen A, Waldmann A, Eisemann N, Katalinic A.Impact of skin cancer screening and secondary prevention campaigns on skin cancer incidence and mortality: A systematic review. J Am Acad Dermatol. 2017;76(1):129-139.e10.

10. Johnson MM, Leachman SA, Aspinwall LG, et al. Skin cancer screening: recommendations for data-driven screening guidelines and a review of the US Preventive Services Task Force controversy. Melanoma Manag. 2017;4(1),13-37.

11. Baughan P, O'Neill B, Fletcher E. Auditing the diagnosis of cancer in primary care: the experience in Scotland. British Journal of Cancer. 2009;101:S87-S91



For peer review only - http://bmjopen.bmj.com/site/about/guidelines.xhtml

12. Kebble S, Abel GA, Saunders CL, MacPhail S, Walter FM, Neal RD, et al. Variation in promptness of presentation among 10,297 patients subsequently diagnosed with one of 18 cancers: Evidence from a national audit of cancer diagnosis in primary care. Int J Cancer, 2014;135(5):1220-8.

13. Walter FM, Birt L, Cavers D. et al. 'This isn't what mine looked like': a qualitative study of symptom appraisal and help seeking in people recently diagnosed with melanoma. BMJ Open 2014; 4(7) e005566.

14. Cancer Research UK website. Available from: http://www.cancerresearchuk.org/health-professional/early-diagnosis-activities/be-clear-on-cancer/skin-cancer-campaign (accessed 6th May 2017).

15. Senior K. Smart phones: new clinical tools in oncology? The Lancet Oncology. 2011;12(5):429-30.

16. Hamidi R, Peng D, Cockburn M. Efficacy of skin self-examination for the early detection of melanoma. Int J Dermatol. 2010; 49(2): 126-34.

17. Carli P, De Giorgi V,Palli D et al. Dermatologist detection and skin self-examination are associated with thinner melanomas: results from a survey of the Italian Multidisciplinary Group on Melanoma. Arch Dermatol. 2003; 139(5):607-12.

18. Korner A, Drapeau M, Thombs BD, et al. Barriers and facilitators of adherence to medical advice on skin self-examination during melanoma follow-up care. BMC Dermatol 2013; 13:3.

19. Murchie P, Allan JL, Brant W, Dennis M, Hall S, Masthoff J, et al. Total skin self-examination at home for people treated for cutaneous melanoma: development and pilot of a digital intervention. BMJ Open. 2015;5(8):e007993.

20. Craig P, Dieppe P, Macintyre S, Michie S, Nazareth I, Petticrew M. Developing and evaluating complex interventions: the new Medical Research Council guidance. BMJ 2008; 337: a1655–a1655

21. Usher-Smith JA, Kassianos A, Emery JD, Abel GA, Teoh Z, Hall S, Neal RD, Murchie P, Walter FM. Identifying people at higher risk of melanoma across the UK: a primary care based electronic survey. Brit J Dermatol. 2017 Apr;176(4):939-948.

22. Williams LH, Shors AR, Barlow WE, Solomon C, White E. Identifying Persons at Highest Risk of Melanoma Using Self-Assessed Risk Factors. J Clin Exp Dermatol Res 2011;2:pii:1000129, doi:10.4172/2155-9554.1000129.

23. Kassianos, A. P., Emery, J. D., Murchie, P., & Walter, F. M. (2015). Smartphone applications for melanoma detection by community, patient and generalist clinician users: a review. British Journal of Dermatology (6), 1507-1518.

24. Braun V, Clarke V. Using thematic analysis in psychology. Qual Res Psychol 2006;3:77–101.

25. Walter FM, Rubin G, Bankhead C, Morris HC, Hall N, Mills K, Dobson C, Rintoul RC, Hamilton W, Emery J. Symptoms and other factors associated with time to diagnosis and stage of lung cancer: a prospective cohort study. Brit J Cancer 2015; 112 Suppl 1:S6-13.

26. Walter FM, Emery JD, Mendonca S, Hall N, Morris HC, Mills K, Dobson C, Bankhead C, Johnson M, Abel GA, Rutter MD, Hamilton W, Rubin GP. Symptoms and patient factors



For peer review only - http://bmjopen.bmj.com/site/about/guidelines.xhtml

associated with longer time to diagnosis for colorectal cancer: results from a prospective cohort study. Brit J Cancer 2016; 115(5): 533-541.

27. Walter FM, Mills K, Mendonca S, Abel G, Basu B, Carroll N, Hamilton W, Rubin GP, Emery JD. Pancreatic cancer: a prospective cohort study of symptoms and patient factors associated with diagnostic intervals and stage at diagnosis. Lancet Gastroenterology & Hepatology, October 4, 2016 http://dx.doi.org/10.1016/S2468-1253(16)30079-6

28. Glanz K, Gelle AC, Shigaki D, Maddock JE, Isnec MR. A randomized trial of skin cancer prevention in aquatics settings: the Pool Cool program. Health Psychol. 2002; 21(6):579-587.

29. Manne S, Lesin S. Prevalence and correlates of sun protection and skin self-examination practices among cutaneous malignant melanoma survivors. J Behav Med. 2006; 29(5):419-34.

30. Moye MS, King SM, Rice ZP, DeLong LK, Seidler AM, Veledar E, Curiel-Lewandrowski C, Chen SC. Effects of total-body digital photography on cancer worry in patients with atypical mole syndrome. JAMA Dermatol. 2015; 151(2):137-43.

31. Lerman C, Trock B, Rimer BK, Jepson C, Brody D, Boyce A. Psychological side effects of breast cancer screening. Health Psychol. 1991;10(4):259-267.

32. Smith S, Fielding S, Murchie P, Johnston M, Wyke S, Powell R, et al. Reducing the time before consulting with symptoms of lung cancer: a randomised controlled trial in primary care. Br J Gen Pract. 2013;63(606):47-54. Epub 2013/01/23.

33. Smith SM, Murchie P, Devereux G, Johnston M, Lee AJ, Macleod U, et al. Developing a complex intervention to reduce time to presentation with symptoms of lung cancer. Br J Gen Pract. 2012;62(602):e605-15. Epub 2012/09/06.

34. Zigmond AS, Snaith RP. The hospital anxiety and depression scale. ACTA Psychiatr Scand 1983; 67:361-70.

35. Ware, J, Kosinski M, Keller SD. A 12-Item Short-Form Health Survey: construction of scales and preliminary tests of reliability and validity. Med Care 1996; 34(3):220-233.

36. Lyratzopoulos G, Saunders CL, Abel GA, McPhail S, Neal RD, Wardle J, Rubin GP. The relative length of the patient and the primary care interval in patients with 28 common and rarer cancers. Br J Cancer 2015; 112 Suppl:S35-40.

For peer review only



For peer review only - http://bmjopen.bmj.com/site/about/guidelines.xhtml

**Table 1: Piloting the intervention- consumer characteristics**

| | Participants (n=16) |
|---|---|
| ***Risk of Melanoma*** | |
| High risk of Melanoma (treated melanoma) | 8 |
| At above population risk | 5 |
| Unknown risk (relative of high risk participant) | 3 |
| ***Gender*** | |
| Male | 8 |
| Female | 8 |
| ***Age*** | |
| <35 years | 2 |
| 36-44 years | 1 |
| 45-54 years | 3 |
| 55-64 years | 7 |
| 65-74 years | 3 |
| ***Ethnicity*** | |
| White British | 15 |
| White Other | 1 |
| ***Education (highest qualification)*** | |
| GCSE or equivalent* | 1 |
| A Level or equivalent** | 3 |
| Vocational | 2 |
| Undergraduate degree | 3 |
| Postgraduate degree or professional qualification | 7 |
| ***Employment status*** | |
| Student | 1 |
| Work part-time | 5 |
| Work full-time | 4 |
| Homemaker | 1 |
| Retired | 5 |
| ***Index of Multiple Deprivation (IMD) Quintiles*** | |
| Least deprived 1 | 8 |
| 2 | 5 |
| 3 | 2 |
| 4 | 0 |
| Most deprived 5 | 0 |
| Unknown | 1 |

* GCSE- General Certificate of Secondary Education, an academic qualification awarded in a specified subject, generally taken in a number of subjects by pupils aged 14-16 in England & Wales.
** A level, same, generally taken aged 16-18.



For peer review only - http://bmjopen.bmj.com/site/about/guidelines.xhtml

For peer review only

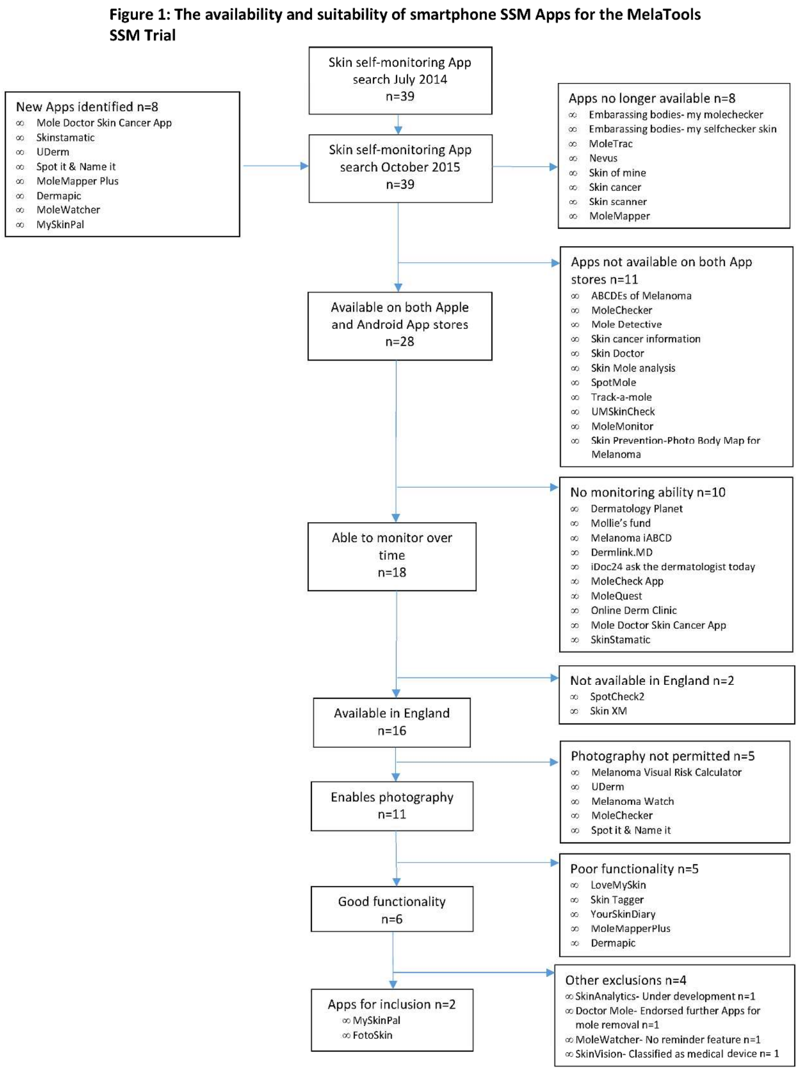


Figure 1: The availability and suitability of smartphone SSM Apps for the MelaTools SSM Trial

209x297mm (300 x 300 DPI)

Figure 2: MelaTools SSM Trial flowchart

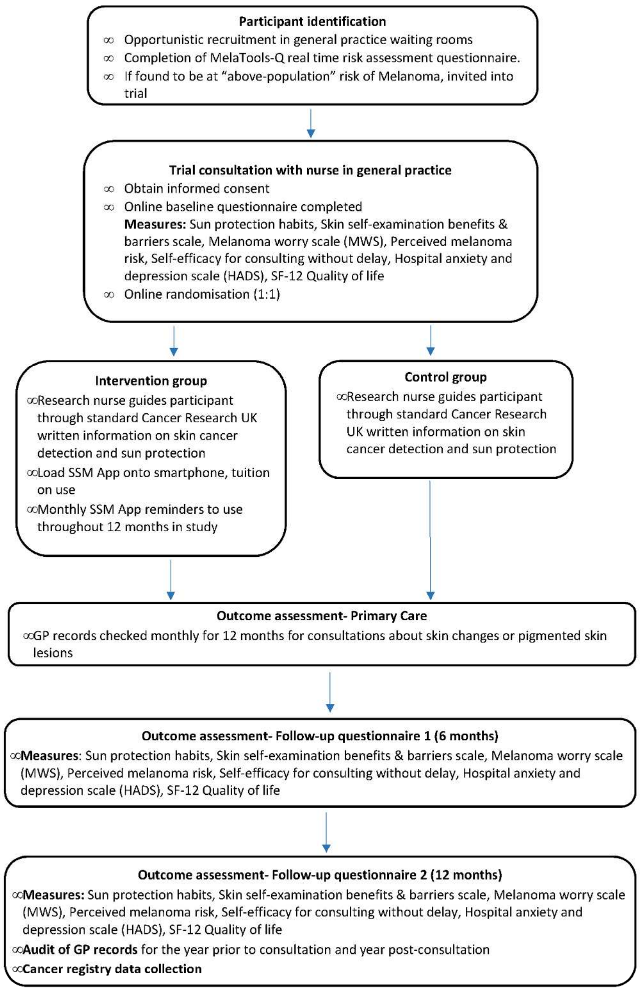


Figure 2: MelaTools SSM Trial flowchart

209x297mm (300 x 300 DPI)

UNIVERSITY OF CAMBRIDGE
Public Health and Primary Care
The Primary Care Unit

MelaTools
improving early diagnosis

NHS
National Institute for Health Research

## MelaTools Skin Self-Monitoring study for primary care patients at higher risk of melanoma

# Consent Form

**Names of Researchers**: Fiona Walter, Katie Mills

Please initial **each** box

1. I confirm that I have read and understand the Participant Information Sheet Version 2, dated 24 June 2016 for the above study and I confirm that the study procedures and information have been explained to me. I have had the opportunity to ask questions and I am satisfied with the answers and explanations provided. ☐
2. I understand that my participation in this study is voluntary and that I am free to withdraw at any time, without giving a reason and without my medical care or legal rights being affected. ☐
3. I understand that sections of my medical notes or information related directly to my participation in this study may be looked at by responsible individuals from the sponsor, regulatory authorities and research personnel where it is relevant to my taking part in research. I give permission for these individuals to have access to my records, including follow-up up to 10 years. ☐
4. I understand that my GP will be informed of my participation in this study and sent details of the study. No medical advice regarding my skin will be issued by the study team and it is my responsibility to contact my GP if I become concerned about a skin change or mole. I understand that the study team will inform my GP if my questionnaire indicates that I am more worried about my health than others. ☐
5. I understand that the researchers in charge of this study may close the study, or stop my participation in it at any time without my consent. ☐
6. I have read and understood my responsibilities for the study. ☐
7. If I am in the group that is invited to download the smartphone App, I understand that the App is accessible to the public on online App stores and is installed onto my own device at my own risk. The study team does not take responsibility for any affect that the App may have on my device or for damage to my device whilst using the App. I am welcome to delete the App from my device at any time. ☐
8. If I am in the group that is invited to download the smartphone App, I understand that the information collected on the App is for my personal use only and the study team will never ask to see this information. ☐
9. I agree to participate in this study. ☐

| Title | Name of participant | Signature | Date |
|---|---|---|---|
| Title | Name of person taking consent | Signature | Date |

*Thank you for completing this form. White copy for research team; yellow copy for participant, blue copy for GP practice.*

For peer review only

For peer review only - http://bmjopen.bmj.com/site/about/guidelines.xhtml

SPIRIT 2013 Checklist: Recommended items to address in a clinical trial protocol and related documents*

| Section/item | Item No | Description | Addressed on page number |
|---|---|---|---|
| **Administrative information** | | | |
| Title | 1 | Descriptive title identifying the study design, population, interventions, and, if applicable, trial acronym | ________1______ |
| Trial registration | 2a | Trial identifier and registry name. If not yet registered, name of intended registry | ________2_____ |
| | 2b | All items from the World Health Organization Trial Registration Data Set | ________n/a____ |
| Protocol version | 3 | Date and version identifier | ________12_____ |
| Funding | 4 | Sources and types of financial, material, and other support | ________12_____ |
| Roles and responsibilities | 5a | Names, affiliations, and roles of protocol contributors | _____1,11______ |
| | 5b | Name and contact information for the trial sponsor | ______11_____ |
| | 5c | Role of study sponsor and funders, if any, in study design; collection, management, analysis, and interpretation of data; writing of the report; and the decision to submit the report for publication, including whether they will have ultimate authority over any of these activities | ______12______ |
| | 5d | Composition, roles, and responsibilities of the coordinating centre, steering committee, endpoint adjudication committee, data management team, and other individuals or groups overseeing the trial, if applicable (see Item 21a for data monitoring committee) | ______12_______ |



For peer review only - http://bmjopen.bmj.com/site/about/guidelines.xhtml

**Introduction**

| | | | |
|---|---|---|---|
| Background and rationale | 6a | Description of research question and justification for undertaking the trial, including summary of relevant studies (published and unpublished) examining benefits and harms for each intervention | ______4-6____ |
| | 6b | Explanation for choice of comparators | ______n/a______ |
| Objectives | 7 | Specific objectives or hypotheses | ______6______ |
| Trial design | 8 | Description of trial design including type of trial (eg, parallel group, crossover, factorial, single group), allocation ratio, and framework (eg, superiority, equivalence, noninferiority, exploratory) | ______7______ |

**Methods: Participants, interventions, and outcomes**

| | | | |
|---|---|---|---|
| Study setting | 9 | Description of study settings (eg, community clinic, academic hospital) and list of countries where data will be collected. Reference to where list of study sites can be obtained | ______7_______ |
| Eligibility criteria | 10 | Inclusion and exclusion criteria for participants. If applicable, eligibility criteria for study centres and individuals who will perform the interventions (eg, surgeons, psychotherapists) | _____7______ |
| Interventions | 11a | Interventions for each group with sufficient detail to allow replication, including how and when they will be administered | ______7_______ |
| | 11b | Criteria for discontinuing or modifying allocated interventions for a given trial participant (eg, drug dose change in response to harms, participant request, or improving/worsening disease) | ___n/a________ |
| | 11c | Strategies to improve adherence to intervention protocols, and any procedures for monitoring adherence (eg, drug tablet return, laboratory tests) | ____n/a______ |
| | 11d | Relevant concomitant care and interventions that are permitted or prohibited during the trial | _____n/a_____ |
| Outcomes | 12 | Primary, secondary, and other outcomes, including the specific measurement variable (eg, systolic blood pressure), analysis metric (eg, change from baseline, final value, time to event), method of aggregation (eg, median, proportion), and time point for each outcome. Explanation of the clinical relevance of chosen efficacy and harm outcomes is strongly recommended | _______8_____ |
| Participant timeline | 13 | Time schedule of enrolment, interventions (including any run-ins and washouts), assessments, and visits for participants. A schematic diagram is highly recommended (see Figure) | ______18______ |



For peer review only - http://bmjopen.bmj.com/site/about/guidelines.xhtml

| | | | |
|---|---|---|---|
| Sample size | 14 | Estimated number of participants needed to achieve study objectives and how it was determined, including clinical and statistical assumptions supporting any sample size calculations | _____10_______ |
| Recruitment | 15 | Strategies for achieving adequate participant enrolment to reach target sample size | _____10______ |
| **Methods: Assignment of interventions (for controlled trials)** | | | |
| Allocation: | | | |
| Sequence generation | 16a | Method of generating the allocation sequence (eg, computer-generated random numbers), and list of any factors for stratification. To reduce predictability of a random sequence, details of any planned restriction (eg, blocking) should be provided in a separate document that is unavailable to those who enrol participants or assign interventions | _____7_______ |
| Allocation concealment mechanism | 16b | Mechanism of implementing the allocation sequence (eg, central telephone; sequentially numbered, opaque, sealed envelopes), describing any steps to conceal the sequence until interventions are assigned | _____n/a______ |
| Implementation | 16c | Who will generate the allocation sequence, who will enrol participants, and who will assign participants to interventions | ______7______ |
| Blinding (masking) | 17a | Who will be blinded after assignment to interventions (eg, trial participants, care providers, outcome assessors, data analysts), and how | ____n/a______ |
| | 17b | If blinded, circumstances under which unblinding is permissible, and procedure for revealing a participant's allocated intervention during the trial | _______n/a___ |
| **Methods: Data collection, management, and analysis** | | | |
| Data collection methods | 18a | Plans for assessment and collection of outcome, baseline, and other trial data, including any related processes to promote data quality (eg, duplicate measurements, training of assessors) and a description of study instruments (eg, questionnaires, laboratory tests) along with their reliability and validity, if known. Reference to where data collection forms can be found, if not in the protocol | ______8-9____ |
| | 18b | Plans to promote participant retention and complete follow-up, including list of any outcome data to be collected for participants who discontinue or deviate from intervention protocols | ____9________ |



For peer review only - http://bmjopen.bmj.com/site/about/guidelines.xhtml

| | | | |
|---|---|---|---|
| Data management | 19 | Plans for data entry, coding, security, and storage, including any related processes to promote data quality (eg, double data entry; range checks for data values). Reference to where details of data management procedures can be found, if not in the protocol | ________10____ |
| Statistical methods | 20a | Statistical methods for analysing primary and secondary outcomes. Reference to where other details of the statistical analysis plan can be found, if not in the protocol | ___9_________ |
| | 20b | Methods for any additional analyses (eg, subgroup and adjusted analyses) | _______10____ |
| | 20c | Definition of analysis population relating to protocol non-adherence (eg, as randomised analysis), and any statistical methods to handle missing data (eg, multiple imputation) | ___10______ |
| **Methods: Monitoring** | | | |
| Data monitoring | 21a | Composition of data monitoring committee (DMC); summary of its role and reporting structure; statement of whether it is independent from the sponsor and competing interests; and reference to where further details about its charter can be found, if not in the protocol. Alternatively, an explanation of why a DMC is not needed | ___10_______ |
| | 21b | Description of any interim analyses and stopping guidelines, including who will have access to these interim results and make the final decision to terminate the trial | ______n/a_____ |
| Harms | 22 | Plans for collecting, assessing, reporting, and managing solicited and spontaneously reported adverse events and other unintended effects of trial interventions or trial conduct | _______n/a_____ |
| Auditing | 23 | Frequency and procedures for auditing trial conduct, if any, and whether the process will be independent from investigators and the sponsor | ______n/a_____ |
| **Ethics and dissemination** | | | |
| Research ethics approval | 24 | Plans for seeking research ethics committee/institutional review board (REC/IRB) approval | __11________ |
| Protocol amendments | 25 | Plans for communicating important protocol modifications (eg, changes to eligibility criteria, outcomes, analyses) to relevant parties (eg, investigators, REC/IRBs, trial participants, trial registries, journals, regulators) | ___11______ |



For peer review only - http://bmjopen.bmj.com/site/about/guidelines.xhtml

| | | | |
|---|---|---|---|
| Consent or assent | 26a | Who will obtain informed consent or assent from potential trial participants or authorised surrogates, and how (see Item 32) | ______6______ |
| | 26b | Additional consent provisions for collection and use of participant data and biological specimens in ancillary studies, if applicable | ___n/a______ |
| Confidentiality | 27 | How personal information about potential and enrolled participants will be collected, shared, and maintained in order to protect confidentiality before, during, and after the trial | _____8_______ |
| Declaration of interests | 28 | Financial and other competing interests for principal investigators for the overall trial and each study site | ___11________ |
| Access to data | 29 | Statement of who will have access to the final trial dataset, and disclosure of contractual agreements that limit such access for investigators | _____12_____ |
| Ancillary and post-trial care | 30 | Provisions, if any, for ancillary and post-trial care, and for compensation to those who suffer harm from trial participation | ___n/a_______ |
| Dissemination policy | 31a | Plans for investigators and sponsor to communicate trial results to participants, healthcare professionals, the public, and other relevant groups (eg, via publication, reporting in results databases, or other data sharing arrangements), including any publication restrictions | ___11_______ |
| | 31b | Authorship eligibility guidelines and any intended use of professional writers | ___12_______ |
| | 31c | Plans, if any, for granting public access to the full protocol, participant-level dataset, and statistical code | ___n/a______ |
| **Appendices** | | | |
| Informed consent materials | 32 | Model consent form and other related documentation given to participants and authorised surrogates | _supplementary file 1_____ |
| Biological specimens | 33 | Plans for collection, laboratory evaluation, and storage of biological specimens for genetic or molecular analysis in the current trial and for future use in ancillary studies, if applicable | ___n/a_______ |

*It is strongly recommended that this checklist be read in conjunction with the SPIRIT 2013 Explanation & Elaboration for important clarification on the items. Amendments to the protocol should be tracked and dated. The SPIRIT checklist is copyrighted by the SPIRIT Group under the Creative Commons "Attribution-NonCommercial-NoDerivs 3.0 Unported" license.



For peer review only - http://bmjopen.bmj.com/site/about/guidelines.xhtml

BMJ Open

# Protocol for the MelaTools Skin Self-Monitoring Trial: a phase II randomised controlled trial of an intervention for primary care patients at higher risk of melanoma

| | |
|---|---|
| Journal: | *BMJ Open* |
| Manuscript ID | bmjopen-2017-017934.R2 |
| Article Type: | Protocol |
| Date Submitted by the Author: | 02-Oct-2017 |
| Complete List of Authors: | Mills, Katie; University of Cambridge, Public Health & Primary Care<br>Emery, Jon; University of Melbourne, General Practice and Primary Care Academic Centre<br>Lantaff, Rebecca; University of Cambridge, Public Health & Primary Care<br>Radford, Michael; University of Cambridge, Public Health & Primary Care<br>Pannebakker, Merel; University of Cambridge, Public Health & Primary Care<br>Hall, Per; Cambridge University Hospitals NHS Foundation Trust<br>Burrows, Nigel; Cambridge University Hospitals NHS Foundation Trust<br>Williams, Kate; University of Cambridge, Public Health & Primary Care<br>Saunders, Catherine; University of Cambridge, Cambridge Centre for Health Services Research<br>Murchie, Peter; University of Aberdeen, Division of Applied Health Science<br>Walter, Fiona; University of Cambridge, Dept of Public Health and Primary Care |
| <b>Primary Subject Heading</b>: | General practice / Family practice |
| Secondary Subject Heading: | Dermatology |
| Keywords: | melanoma, skin cancer, randomised controlled trial, risk-stratified, PRIMARY CARE |
| | |

SCHOLARONE™ Manuscripts

# Protocol for the MelaTools Skin Self-Monitoring Trial: a phase II randomised controlled trial of an intervention for primary care patients at higher risk of melanoma

Mills K[1], Emery JD[2,1], Lantaff R[1], Radford M[3], Pannebakker MM[1], Hall P[4], Burrows NP[4], Williams KM[1], Saunders CL[1], Murchie P[5], Walter FM[1,2]

[1] The Primary Care Unit, Department of Public Health and Primary Care, University of Cambridge, CB1 8SR, UK
[2] Department of General Practice and the Centre for Cancer Research, Faculty of Medicine, Dentistry and Health Science, Victorian Comprehensive Cancer Centre, University of Melbourne, Australia
[3] Tennyson House Surgery, Chelmsford CM1 4HW, UK
[4] Cambridge University Hospitals NHS Foundation Trust, Hills Road, Cambridge CB2 0QQ, UK
[5] Division of Applied Health Science, Centre of Academic Primary Care, University of Aberdeen, Polwarth Building, Foresterhill, Aberdeen AB25 2ZD, UK

**Correspondence to:** Katie Mills: ko298@medschl.cam.ac.uk

**Key words:** melanoma, skin cancer, randomised controlled trial, risk-stratified, primary care

**Word Count**: 3876
2 figures, 1 table

For peer review only



For peer review only - http://bmjopen.bmj.com/site/about/guidelines.xhtml

**ABSTRACT**

**Introduction**

Melanoma is the fifth commonest cancer in the UK. Incidence rates have quadrupled over the last 30 years and continue to rise, especially among younger people. As routine screening of the general population is not currently recommended in the UK, a focus on secondary prevention through early detection and prompt treatment in individuals at increased risk of melanoma could make an important contribution to improve melanoma outcomes. This paper describes the protocol for a phase II, multisite, randomised controlled trial, in the primary care setting, for patients at increased risk of melanoma. A skin self-monitoring smartphone 'App' was used to improve symptom appraisal and encourage help-seeking in primary care, thereby promoting early presentation with skin changes suspicious of melanoma.

**Methods and analysis**

We aim to recruit 200 participants from general practice waiting rooms in the East of England. Eligible patients are those identified at higher melanoma risk (using a real-time risk assessment tool), without a personal history of melanoma, aged 18 to 75 years. Participants will be invited to a primary care nurse consultation, and randomised to the Intervention group (standard written advice on skin cancer detection and sun protection, loading of a skin self-monitoring 'App' onto the participant's smartphone, and instructions on use including self-monitoring reminders) or Control group (standard written advice alone). The primary outcomes are consultation rates for changes to a pigmented skin lesion, and the patient interval (time from first noticing a skin change to consultation). Secondary outcomes include patient sun-protection behaviours, psychosocial outcomes, and measures of trial feasibility and acceptability.

**Ethics and dissemination**

NHS ethical approval has been obtained from Cambridgeshire and Hertfordshire research ethics committee (REC reference 16/EE/0248). The findings from the MelaTools SSM Trial will be disseminated widely through peer-reviewed publications and scientific conferences.

**Trial registration number:** ISCTRN registry, ISCTRN16061621

For peer review only



For peer review only - http://bmjopen.bmj.com/site/about/guidelines.xhtml

**Strengths and limitations of this study**

- The MelaTools SSM Trial is among the first, in the UK or internationally, to risk stratify the primary care population in order to target an intervention to improve timeliness of melanoma detection.

- It uses digital technology to encourage people at higher risk of melanoma to self-monitor their skin and present to their physician with any concerns.

- The trial aims to establish the feasibility and acceptability of undertaking skin self-monitoring among people at higher risk of melanoma; it will also report on consultation rates and includes 6- and 12-month participant follow-up.

- As this is a feasibility trial the main limitation is that the small sample size will limit meaningful interpretation of the clinical outcomes; furthermore, the sample may not be fully representative of the UK adult population as it excludes people who do not own a smartphone, and those with physical disorders severe enough to hamper smartphone use.

- Information from this trial will enable planning of a larger phase III trial to further assess the impact of the use of a smartphone App to encourage skin self-monitoring on clinical outcomes for melanoma.


For peer review only - http://bmjopen.bmj.com/site/about/guidelines.xhtml

## INTRODUCTION

Malignant melanoma is the leading cause of skin cancer deaths in the UK with 2,459 in 2014 (1). Melanoma skin cancer incidence has quadrupled over the last 30 years, and continues to rise (2). Although melanoma is more common with increasing age, around half of melanoma in the UK each year are diagnosed in people aged less than 65 (3, 4). Risk factors include fair skin, family history of melanoma, multiple naevi, and sun damage. Melanoma is associated with significant morbidity, and the thickness of the lesion at diagnosis is the most important prognostic factor: stage 1 disease has 5-year survival rates of over 95%, compared to less than 25% for stage 4 disease (5). The UK has lower 1- and 5-year melanoma survival rates than comparable countries in Europe (6). Diagnostic delays are thought to contribute to this, and there is evidence of avoidable delay (7). Evidence from the SCREEN project in Germany, conducted between 2003 and 2004, suggested that population screening may have an impact on melanoma incidence and 5-year mortality (8, 9). However, this evidence is controversial, and routine screening of the general population is not currently recommended anywhere worldwide, although some countries (Australia, New Zealand, Germany, the Netherlands and the UK) recommend regular skin checks and/or self-examination for certain subsets of patients at increased risk of melanoma (10). Therefore, a focus on targeted population screening could make an important contribution to melanoma outcomes through early, timely detection and prompt treatment.

Patient pathways to presentation and management in primary care are key determinants in cancer outcomes. When compared with people diagnosed with other cancers, those diagnosed with melanoma have the second longest median time between first noticing a symptom and presenting to primary care (patient interval) (11, 12). The patient interval could potentially be reduced by providing patients with clear information on the signs and symptoms of melanoma and guidance on monitoring skin changes (13), through community campaigns such as the Be Clear on Cancer skin cancer campaign (14). More targeted approaches could focus on individuals at higher risk.

Smartphones offer considerable potential to promote earlier presentation by people with cancer, including melanoma (15). More than 70% of the UK adult population now own a smartphone (Techtracker), and there are many smartphone applications (‘Apps’) focusing on health issues, presenting new opportunities for risk assessment, symptom appraisal, monitoring symptoms and signs over time, and cues to seek professional advice. Furthermore, up to 75% of melanomas are detected by people or their family/friends, rather than health care professionals. To maximise the effectiveness of skin self-monitoring (SSM) the person performing the examination should be able to identify skin changes and features of skin lesions which could indicate melanoma, yet recent studies have demonstrated that suspicious signs of skin cancer may not be widely known (16,13). Important international evidence has shown that it is possible to educate patients and the public: newly diagnosed Italian melanoma patients who performed self-skin examination were found to have thinner tumours (17), and Australian melanoma patients were found to adhere to medical advice on skin self-examination during follow-up care (18). A recent Scottish study has demonstrated melanoma patients are prepared to use digital technology



For peer review only - http://bmjopen.bmj.com/site/about/guidelines.xhtml

to support them in conducting SSM during follow-up (19). We therefore set out to explore using mobile technology for SSM among people at higher risk of melanoma in the primary care setting to encourage timely consultation for possible melanoma.

**Preliminary Phase I research**

Guided by the Medical Research Council (MRC) framework on developing and evaluating complex interventions (20), three Phase I studies were conducted to: (i) assess the feasibility and acceptability of defining a population at higher risk of melanoma from UK general practice; (ii) review the availability of suitable smartphone SSM Apps, and (iii) pilot the intervention.

***(i) Assessing the feasibility and acceptability of defining a population at higher risk of melanoma from UK general practice***

The MelaTools-Q Study findings have already been reported (21). Briefly, 7,742 patients (86% of those approached) were recruited from the waiting rooms of 22 general practices in three UK regions: Eastern England, Northeast Scotland and North Wales. Using tablet computers, they completed an electronic questionnaire incorporating the Williams melanoma risk prediction model (22), including items relating to 7 risk factors: gender, age, natural hair colour at age 15, number of severe sunburns aged 2-18, prior non-melanoma skin cancer, number of raised moles on both arms, and density of freckles on both arms before age 20. The study showed that, after weighting to the age and sex distribution, the lower and upper quartile cut-offs used by Williams *et al.*, 25 and 34, would allow between 4% and 20% of the population to be identified as higher risk, and those groups would contain 30% and 60%, respectively of individuals who would develop melanoma. Therefore, real-time risk assessment for melanoma in UK primary care is both feasible and acceptable.

***(ii) Reviewing the availability of suitable smartphone SSM Apps***

Our initial review, conducted in July 2014 identified 39 smartphone Apps available from Apple and Android App online stores (23). However, when the same search strategy was re-run in September 2015, 8 new SSM Apps were identified, while 8 of the previously identified SSM Apps were no longer available.  Among the 39 newly-identified SSM Apps, 11 were not available in both Apple and Android App stores; 10 did not include a monitoring function; 5 did not include photography of skin lesions; 5 had poor functionality; 2 were not available in England; 2 were classified as medical devices; one was under development, and one had no reminder feature, deemed vital for monitoring functionality. Two SSM Apps were considered potentially most suitable to include in the intervention (see Figure 1).

***(iii) Piloting the intervention***

Qualitative research, using focus groups and interviews, was conducted with individuals at increased risk of melanoma to provide in-depth understanding of consumer views on the usefulness and usability of the two selected SSM Apps. Eight people at increased risk of melanoma were recruited for two focus groups from a local melanoma patient support group; three of their relatives also took part.  People were also recruited in the waiting rooms of two suburban general practices and completed the Melatools-Q risk assessment tool (21). Five identified to be at increased risk of melanoma were invited to participate in two other focus groups. A



For peer review only - http://bmjopen.bmj.com/site/about/guidelines.xhtml

total of 16 participants tested at least one of the SSM Apps. The participant characteristics are shown in Table 1.

The four focus groups were conducted by an experienced qualitative researcher (KM) and research assistant (RL), either at the recruiting general practice or university premises; each lasted approximately 90 minutes, was audio-recorded, and the recordings transcribed verbatim. Each participant was initially helped to load the two SSM Apps on their smartphones, and given time to familiarise themselves with their use. Thematic analysis (24) showed that, overall, participants in all four groups and at all strata of increased risk of melanoma felt that using a SSM App would be helpful and achievable. Many felt that using a SSM App would enhance their current skin self-monitoring habits: *"I look at the moles and I just keep an eye on them" (Male, above-population risk, group 3).* Many also felt that using a SSM App could help reduce their worry about developing skin cancer: *"cos my dad had cancer recently so [my mother] says we're at a higher risk, so she goes on about that" (Female, above-population risk, group 4).* Some felt that using a SSM App would guide their help-seeking behaviour for skin changes or moles: *"It's that knowledge, if it's, you know, three months, whatever it is, if you can actually say look there is a visible change then you're getting a bit of information that they probably could do with…you've got physical evidence, and then the confidence to go to…" (Male, above-population risk, group 4).* Several also commented on additional features on smartphones which might help prevent development of future melanomas: *"I've got a UV index on my phone so I can always check what that is, so putting sun cream on." (Male, high risk, group 2).*

The five participants recruited via primary care were invited to complete a diary one and two months later, and then participate in a telephone interview after the third month. Diary records and thematic analysis of the interviews (as above) confirmed that participants used many features of both SSM Apps, including: taking photographs, mapping these to body parts, comparing photographs over time, and using the analysis function (available in only one of the Apps). There was unanimous agreement between participants about the App which was easier to use (MySkinPal), and most reported that they would continue to use it after the study, particularly as they found the App's notifications the most efficient method to prompt them to complete future skin self-monitoring. Despite this, several also reported that they found taking photographs quite challenging, and that they had to recruit a family member to assist them in taking photographs to compare skin changes over time. Furthermore, while regularly self-monitoring their skin during the study, most participants reported worrying about skin cancer 'sometimes', seeking further information about skin changes or moles via the internet, speaking to friends and family, and visiting their general practice.

In conclusion, this preliminary Phase I work enabled consumer choice of the more user-friendly SSM App, resulted in modifications to the language used in the trial consultation, and led to the development of the MelaTools SSM Trial.

The trial aims to assess the effect of using a SSM App compared with standard information about detecting skin cancer on consultation rates and the patient



For peer review only - http://bmjopen.bmj.com/site/about/guidelines.xhtml

interval (the time from first noticing a skin change/mole to consultation with a primary care healthcare professional) among patients at increased risk of melanoma, identified via primary care. In addition, to obtain preliminary estimates of effect across a range of outcome measures to inform selection of the primary outcome for a definitive phase III trial. We hypothesise that that primary care patients at higher risk of melanoma will seek help more rapidly after noticing skin changes when using a SSM App compared with using standard written information.

## METHODS AND ANALYSIS

### Study design and setting

The phase II trial is a multi-site randomised controlled trial (Figure 2), set in 11 general practices across Eastern England. Those who meet the eligibility criteria and consent to participate are randomised 1:1 into either the control or intervention group. Randomisation is being performed using an online system provided by the Clinical Trials Unit based at Kings College London.

### Study participants

#### *Eligibility criteria*

Eligible participants are individuals aged between 18 and 75 years who own a smartphone (Apple or Android), and, on completion of the Melatools-Q risk assessment tool (21), are found to be at increased risk of melanoma. Participants are able to read and write English and to give informed consent.

#### *Exclusion criteria*

Exclusion criteria are previous diagnosis of melanoma, severe psychiatric or cognitive disorders, or a physical disorder severe enough to inhibit the use of a smartphone.

#### *Participant recruitment*

Potentially eligible individuals are approached by research nurses or research assistants in GP waiting areas at different times of the day and different days of the week, in order to ensure that a broad range of ages, gender, and educational level are approached. Those meeting initial screening criteria are given a tablet computer that includes a short version of the participant information sheet and an online consent form agreeing to have their risk assessed and be considered for the trial. They complete the MelaTools Q study risk assessment tool (21) which stratifies respondents into 'population risk' (majority) and 'increased risk', using a cut-off score in the Williams model of 25. People at 'population risk' are thanked and given the Cancer Research UK leaflets on risk factors of melanoma and melanoma detection advice. People at increased risk are invited to participate in the trial, and given the full-length trial Participant Information Sheet and an appointment time within 14 days for the primary care nurse trial consultation. Individuals not wishing to take part are asked their reason for declining.

#### *Control and Intervention groups and randomisation*

Informed written consent is obtained from all participants at the start of the trial consultation (supplementary file 1). After completion of a baseline questionnaire, the trial nurse guides the participant through the Cancer Research UK leaflets on "Be SunSmart cut your cancer risk" and "Skin cancer - How to spot the signs and



For peer review only - http://bmjopen.bmj.com/site/about/guidelines.xhtml

symptoms". Online randomisation is then performed: those randomised to the control group are thanked for their participation and both groups are given the Cancer Research UK leaflets to take home.

***Intervention group***

Participants randomised to the intervention group are assisted with loading the skin self-monitoring App onto their smartphone by the trial nurse, who continues by giving instructions on its use, supported by written instruction sheets. The core functions of the App, MySkinPal, include: full body scan, body diagrams, photo source and browser, information on the signs of melanoma, regular reminders, ability to select moles to share with a doctor, and motivation to use including awards/achievements. The nurse ensures the App's monthly reminder notification is switched on to prompt each participant regularly. Finally, each intervention participant is given an Apple Apps store/Google Play voucher (up to £5) to pay for the App.

**Outcomes and measures**

The primary outcomes of this trial are *consultation rates* for pigmented skin lesions, and the *patient interval*. *Consultation rates* for changes to an existing pigmented skin lesion or concerns about a new pigmented skin lesion for the 12 months prior to the trial and the 12 months after the trial consultation will be collected via searching general practice electronic medical records using a clinical notes audit for every participant. The *patient interval* is the time from first noticing a skin change to first consultation. These data will be collected monthly by searches of general practice electronic medical records for all skin changes or pigmented skin lesions presented to their GP or practice nurse during the 12 months following the trial consultation. When a participant consults about a pigmented skin lesion, they receive a Skin Questionnaire to complete. This skin questionnaire uses the Symptom Study instrument modified for melanoma symptoms. The instrument is a participant-completed questionnaire collecting data on symptoms and their duration prior to consultation, validated in prospective studies examining symptom and patient factors associated with longer time to diagnosis for colorectal, lung and pancreatic cancer (25-27). The skin questionnaire comprises 10 items starting with a free text response to 'What was the first skin change or mole you noticed that made you think something might be wrong?' Each item includes the sub-items: 'When did you first notice this?' and 'When did you first tell your GP or nurse?'

Secondary measures and outcomes (measured at baseline, 6- and 12-months) include:

(1) *Demographics and clinical variables:* age, gender, marital status, postcode, highest education level, occupation, past history of skin cancer (melanoma, Squamous Cell Carcinoma, Basal Cell Carcinoma), skin and hair type (density of freckles on arms before age 20, natural hair colour at age 15, number of severe sunburns aged 2-18), number of raised moles on both arms, measured at baseline only (21,22), collected as part of the baseline eligibility assessment;



For peer review only - http://bmjopen.bmj.com/site/about/guidelines.xhtml

(2) *Sun protection habits scale*: developed by Glanz et al in the US for a multicomponent skin cancer prevention programme, this comprises 5 items measured using a 4 point Likert scale, and relating to use of sun protection, sun and sunbed habits, and episodes of sunburn in the previous year (28), as we hypothesise that the App reminders will not only prompt people but also reinforce the messages on sun protection;

(3) *Skin Self-Examination benefits and barriers scale*: validated by Manne and Lessin in the US among melanoma survivors, and developed from previous work on mammography and family members of patients with colorectal cancer (29). The benefits scale has seven items (α=.71) and the barriers scale has ten items (α=.74);

(4) *Melanoma Worry Scale (MWS):* validated by Moye et al in the US (30), an adapted from the Breast Cancer Worry Scale (31), this measure comprises four items, scored 1 to 4, with possible scores ranging from 4 to 17, and higher scores indicating higher levels of worry;

(5) *Perceived Melanoma Risk*: drawn from Manne and Lessin's measures (29), these two items have been widely used for melanoma and other cancer risk assessments to assess estimated percent risk of developing melanoma, and perceived risk compared with a person of the same age (relative risk);

(6) *Self-Efficacy for consulting without delay*: A 10-item self-completed scale summed to score 10-100, was used in a primary care trial for lung symptoms, and showed good internal reliability (Cronbach α =0.85) (32, 33). It has been adapted for this trial, and reduced to 8 items, e.g. 'How confident are you that you can make an appointment to see a doctor when…… you can't get an appointment with your usual doctor?';

(7) *Hospital anxiety and depression scale (HADS):* This 14-item self-completed scale has been widely used to measure distress and has been extensively validated and shown to perform well in a wide range of populations (mean Cronbach α = 0.82; sensitivity and specificity 0.80) (34);

(8) *SF-12 Quality of life scale*: a 12-item version of the SF-36 that is widely used and validated to measure functional health and well-being (35), e.g. 'During the past 4 weeks, have you had any of the following problems with your work or other regular daily activities as a result of your physical health?.....'

Other outcome measures include:

(9) *Trial feasibility and acceptability*, including: data on patient recruitment and attrition rates, reasons for attrition, response rates to Skin Symptom Questionnaires for measuring the patient interval to inform decisions about a future phase III trial.

(10) *Melanoma incidence across participating practices*, to contextualise trial findings and after 5 years. This will be identified through GP electronic medical records and the National Cancer Registration Service.

**Measurement timings and study end points**

At the trial consultation all participants will be fully informed about trial follow-up procedures. The participant-reported measures (numbers 1-8 above) are completed in person at baseline, and online at follow-up 1 (6 months) and follow-up 2 (12 months), with the exception of the skin symptom questionnaire as already



For peer review only - http://bmjopen.bmj.com/site/about/guidelines.xhtml

described, which is sent to participants by postal mail within a month of any consultation for skin changes or pigmented skin lesions.

After trial completion, a final audit of the GP electronic medical records will be run to identify all skin consultations for the 12 months during the trial as well as the previous 12 months. Melanoma incidence findings will also be collected at the end of the 12 month follow up period and at 5 years. These data will not be collected for participants who formally withdraw from the trial.

**Participant data and study management**

All participants are allocated a unique identifying code. Melanoma risk assessment data, baseline questionnaire, and follow up questionnaires data are stored on a custom built database on a secure server hosted by the Outcome Registry Intervention and Operation Network (ORION), Clinical Neurosciences department at the University of Cambridge.

**Sample size consideration**

Based on our recent MelaTools Q study with an identical screening step (response rate of 86%), we anticipate that we will need to approach approximately 2,000 people from 10 general practice waiting rooms for about 1,600 to complete the Melatools Q risk assessment tool (23). About 25%, 400 people, will be identified as increased risk and be eligible to participate in the trial. Based on previous research we would expect approximately 50% of these to attend their trial consultation and undergo randomisation in order to reach our target of 200 participants.

We will use information on recruitment, attrition, and data completeness, and preliminary estimates of effect sizes where appropriate, to inform the sample size calculation for a future phase III trial.

**Statistical analysis**

All randomised patients will be considered eligible for inclusion in the analysis in accordance with the intention-to-treat analysis principle. As this is a phase II trial, fully describing and characterising the extent and nature of the missing data is an important part of the analysis. For the outcome analysis, appropriate methods for dealing with missing endpoint data will be informed by a blinded review of the data; however, assuming that this is appropriate, we plan a series of extreme case-sensitivity analyses, where missing data will be replaced with the $2.5^{th}$ and $97.5^{th}$ centiles of the non-missing measured outcomes to assess the maximum potential impact on the results of the trial. The baseline characteristics of the two arms will be described using summary statistics. Possible consent bias will be assessed by comparing demographic and clinical variables of participants against those who declined participation, and possible differential attrition will be assessed by comparing baseline characteristics of those who withdraw or die against those who remain in the trial. In addition we will analyse the missing data to understand the potential impact on the required sample size, and to assess whether and where possible bias may arise, and therefore be minimised, in the future design for the efficacy phase III trial. We will describe the amount (percentage) of missing data in all outcome measures at baseline and follow up, and evaluate the relationship



For peer review only - http://bmjopen.bmj.com/site/about/guidelines.xhtml

between missing data and treatment assignment using unadjusted chi-squared tests. We will describe whether missing data occurs differentially between outcomes. These comparisons will be performed using a two sample t-test (or non-parametric equivalent) for continuous variables and chi-square test for categorical variables.

The primary analysis will be a comparison between the two groups on the consultation rate for skin changes/pigmented skin lesion using a Poisson regression model. Comparisons between groups on binary secondary endpoints will be performed using logistic regression. Comparisons between groups on continuous secondary endpoints will be undertaken using a linear model that includes the baseline value where applicable. A linear model may not be appropriate for the secondary outcome Patient Interval, as this is expected to be right skewed (36). We will explore the nature and extent of the skewness and analyse Patient Interval using a linear model if possible, adopting other approaches as appropriate, including transformation or categorisation of the Patient Interval, or methods for statistical inference based on bootstrap resampling. The analyses performed on the primary and secondary endpoints will be repeated adjusting for additional baseline covariates as part of a sensitivity analysis. Point estimates of the intervention effect will be presented with 95% confidence intervals and two-sided p-values. Unadjusted p-values from secondary analyses will be interpreted in proper context and be clearly labelled.

## ETHICS AND DISSEMINATION

### Ethical approval

This trial is sponsored by the School of Clinical Medicine at the University of Cambridge. Ethical approval for the MelaTools SSM trial was granted on 11th July 2016 by the Cambridgeshire and Hertfordshire NRES research ethics committee (reference number 16/EE/0248). Health Research Authority approval (HRA) was granted on 22nd July 2016. R&D approvals have been obtained from the relevant Trusts. All substantial amendments are approved by the NHS Research Ethics committee responsible for the trial, in addition to approval by HRA and NHS R&D. Investigators are kept up to date with relevant changes via regular management group meetings.

### Data monitoring

Due to the non-medicinal and low-risk nature of the trial a data monitoring committee will not be needed. The trial steering committee (the CI, collaborators and researchers, statistician and PPIE representatives) will meet 6 monthly from the start of the trial and will monitor trial progress, approve a data analysis plan, and will ensure the trial runs in accordance with the protocol and applicable standard operating procedures. The CI will take responsibility for data monitoring and ethics, and will be responsible for communicating important protocol modifications to relevant parties.

### Expected findings, implications and dissemination

Twelve-month follow-up will complete in January 2018. We expect that the trial will prove to be both feasible and acceptable to participants without causing them



For peer review only - http://bmjopen.bmj.com/site/about/guidelines.xhtml

significant anxiety or harm. We do not expect to be able to draw any significant clinical conclusions from this small trial, therefore we expect to be use these findings to support a funding application for a larger Phase III trial aiming to be sufficiently powered to enable evaluation of clinical outcomes. We are also interested in further work exploring the usefulness of different apps for different groups of consumers.

We will submit the findings of our research for publication in highly cited and open access peer-reviewed journals, and the findings will also be presented at national and international conferences. We will also provide a summary of our findings on the MelaTools programme website MelaTools.org.

For peer review only

**Acknowledgements**

The authors are grateful to the patients and general practices who contributed to the pilot study. The authors would also like to thank the MelaTools Steering Committee for their scientific support and comments throughout the design and set-up of the trial: Pippa Corrie, Katharine Acland, Juliet Usher-Smith, Ed Wilson, Margaret Johnson, Simon Rodwell and Patricia Fairbrother. This study is supported by the UK Clinical Research Collaboration-registered King's Clinical Trials Unit at King's Health Partners, which is part-funded by the NIHR Biomedical Research Centre for Mental Health at South London and Maudsley NHS Foundation Trust and King's College London and the NIHR Evaluation, Trials and Studies Coordinating Centre.

**Contributors**

FMW and JE developed the initial idea for the trial, and all authors (except MP) have assisted with the development of the protocol, study design and refinement of study materials. PM and JE provided experience of primary care trials, and NB and PH provided clinical expertise. MR led the 2015 SSM Apps review. KM conducted the focus groups and interviews, and led trial preparations with RL and MP, and supported by trials and governance expertise from KW. CS is leading the statistical analysis. KM and FMW wrote the manuscript and revised it in response to critical revisions from all authors. FMW is the guarantor of the study.
The International Committee of Medical Journal Editors (ICMJE) authorship eligibility guidelines have been followed and no professional writers have been used.

**Funding**

This work was supported by FMW's Clinician Scientist award (RG 68235) from the National Institute for Health Research (NIHR). The views expressed in this publication are those of the authors and not necessarily those of the National Health Service, the NIHR or the Department of Health. The funding source has no role in the design of this study, the interpretation of data, or the decision to submit results.
The paper also presents independent research funded/supported by the National Institute for Health Research (NIHR) Collaboration for Leadership in Applied Health Research & Care (CLAHRC) East of England, at Cambridgeshire and Peterborough NHS Foundation Trust. The views expressed are those of the author(s) and not necessarily those of the NHS, the NIHR or the Department of Health,

**Competing interests**

None declared.

**Ethics approval**

Cambridgeshire and Hertfordshire NHS REC committee, reference 16/EE/0248, protocol version 2 24th June 2016. Requests for the final dataset should be addressed to the corresponding author.

**Trial progress**

The study was registered in the ISRCTN Trials Registry on 17th August 2016, the first participant was recruited on 22nd August 2016, enrolment was completed on 6th January 2017, and the participants continue to be followed up in the study, with an estimated finish date of end January 2018.



For peer review only - http://bmjopen.bmj.com/site/about/guidelines.xhtml

For peer review only

**Open Access**
This is an Open Access article distributed in accordance with the terms of the Creative Commons Attribution (CC BY 4.0) license, which permits others to distribute, remix, adapt and build upon this work, for commercial use, provided the original work is properly cited. See: http://creativecommons.org/licenses/by/4.0/

**Figure Legends**
Figure 1: The availability and suitability of smartphone SSM Apps for the MelaTools SSM Trial
Figure 2: MelaTools SSM Trial flowchart

For peer review only

**REFERENCES**

1. Cancer Research UK website. Available from: http://www.cancerresearchuk.org/health-professional/cancer-statistics/statistics-by-cancer-type/skin-cancer#heading-One (accessed 6th May 2017).

2. Arnold M, Holterhues C, Hollestein LM, et al. Trends in incidence and predictions of cutaneous melanoma across Europe up to 2015. J Eur Acad Dermatol Venereol. 2014; 28:1170-8.

3. Diepgen T, Mahler V. The epidemiology of skin cancer. British Journal of Dermatology. 2002;146(s61):1-6.

4. Balch CM, Gershenwald JE, Soong S-J, Thompson JF, Atkins MB, Byrd DR, et al. Final version of 2009 AJCC melanoma staging and classification. Journal of Clinical Oncology. 2009;27(36):6199-206.

5. Cancer Research UK website. Available from: http://www.cancerresearchuk.org/cancer-info/cancerstats/types/skin/survival/skin-cancer-survival-statistics#one (accessed 6th May 2017).

6. Sant M, Allemani C, Santaquilani M, Knijn A, Marchesi F, Capocaccia R, et al. EUROCARE-4. Survival of cancer patients diagnosed in 1995–1999. Results and commentary. European Journal of Cancer. 2009;45(6):931-91.

7. Abdel-Rahman M, Stockton D, Rachet B, Hakulinen T, Coleman M. What if cancer survival in Britain were the same as in Europe: how many deaths are avoidable? British Journal of Cancer. 2009;101:S115-S24.

8. Breitbart EW, Waldmann A, Nolte S, Capellaro M, Greinert R, Volkmer B, et al. Systematic skin cancer screening in Northern Germany. Journal of the American Academy of Dermatology. 2012;66(2):201-11.

9. Brunssen A, Waldmann A, Eisemann N, Katalinic A.Impact of skin cancer screening and secondary prevention campaigns on skin cancer incidence and mortality: A systematic review. J Am Acad Dermatol. 2017;76(1):129-139.e10.

10. Johnson MM, Leachman SA, Aspinwall LG, et al. Skin cancer screening: recommendations for data-driven screening guidelines and a review of the US Preventive Services Task Force controversy. Melanoma Manag. 2017;4(1),13-37.

11. Baughan P, O'Neill B, Fletcher E. Auditing the diagnosis of cancer in primary care: the experience in Scotland. British Journal of Cancer. 2009;101:S87-S91



For peer review only - http://bmjopen.bmj.com/site/about/guidelines.xhtml

12. Kebble S, Abel GA, Saunders CL, MacPhail S, Walter FM, Neal RD, et al. Variation in promptness of presentation among 10,297 patients subsequently diagnosed with one of 18 cancers: Evidence from a national audit of cancer diagnosis in primary care. Int J Cancer, 2014;135(5):1220-8.

13. Walter FM, Birt L, Cavers D. et al. 'This isn't what mine looked like': a qualitative study of symptom appraisal and help seeking in people recently diagnosed with melanoma. BMJ Open 2014; 4(7) e005566.

14. Cancer Research UK website. Available from: http://www.cancerresearchuk.org/health-professional/early-diagnosis-activities/be-clear-on-cancer/skin-cancer-campaign (accessed 6th May 2017).

15. Senior K. Smart phones: new clinical tools in oncology? The Lancet Oncology. 2011;12(5):429-30.

16. Hamidi R, Peng D, Cockburn M. Efficacy of skin self-examination for the early detection of melanoma. Int J Dermatol. 2010; 49(2): 126-34.

17. Carli P, De Giorgi V,Palli D et al. Dermatologist detection and skin self-examination are associated with thinner melanomas: results from a survey of the Italian Multidisciplinary Group on Melanoma. Arch Dermatol. 2003; 139(5):607-12.

18. Korner A, Drapeau M, Thombs BD, et al. Barriers and facilitators of adherence to medical advice on skin self-examination during melanoma follow-up care. BMC Dermatol 2013; 13:3.

19. Murchie P, Allan JL, Brant W, Dennis M, Hall S, Masthoff J, et al. Total skin self-examination at home for people treated for cutaneous melanoma: development and pilot of a digital intervention. BMJ Open. 2015;5(8):e007993.

20. Craig P, Dieppe P, Macintyre S, Michie S, Nazareth I, Petticrew M. Developing and evaluating complex interventions: the new Medical Research Council guidance. BMJ 2008; 337: a1655–a1655

21. Usher-Smith JA, Kassianos A, Emery JD, Abel GA, Teoh Z, Hall S, Neal RD, Murchie P, Walter FM. Identifying people at higher risk of melanoma across the UK: a primary care based electronic survey. Brit J Dermatol. 2017 Apr;176(4):939-948.

22. Williams LH, Shors AR, Barlow WE, Solomon C, White E. Identifying Persons at Highest Risk of Melanoma Using Self-Assessed Risk Factors. J Clin Exp Dermatol Res 2011;2:pii:1000129, doi:10.4172/2155-9554.1000129.

23. Kassianos, A. P., Emery, J. D., Murchie, P., & Walter, F. M. (2015). Smartphone applications for melanoma detection by community, patient and generalist clinician users: a review. British Journal of Dermatology (6), 1507-1518.

24. Braun V, Clarke V. Using thematic analysis in psychology. Qual Res Psychol 2006;3:77–101.

25. Walter FM, Rubin G, Bankhead C, Morris HC, Hall N, Mills K, Dobson C, Rintoul RC, Hamilton W, Emery J. Symptoms and other factors associated with time to diagnosis and stage of lung cancer: a prospective cohort study. Brit J Cancer 2015; 112 Suppl 1:S6-13.

26. Walter FM, Emery JD, Mendonca S, Hall N, Morris HC, Mills K, Dobson C, Bankhead C, Johnson M, Abel GA, Rutter MD, Hamilton W, Rubin GP. Symptoms and patient factors



For peer review only - http://bmjopen.bmj.com/site/about/guidelines.xhtml

associated with longer time to diagnosis for colorectal cancer: results from a prospective cohort study. Brit J Cancer 2016; 115(5): 533-541.

27. Walter FM, Mills K, Mendonca S, Abel G, Basu B, Carroll N, Hamilton W, Rubin GP, Emery JD. Pancreatic cancer: a prospective cohort study of symptoms and patient factors associated with diagnostic intervals and stage at diagnosis. Lancet Gastroenterology & Hepatology, October 4, 2016 http://dx.doi.org/10.1016/S2468-1253(16)30079-6

28. Glanz K, Gelle AC, Shigaki D, Maddock JE, Isnec MR. A randomized trial of skin cancer prevention in aquatics settings: the Pool Cool program. Health Psychol. 2002; 21(6):579-587.

29. Manne S, Lesin S. Prevalence and correlates of sun protection and skin self-examination practices among cutaneous malignant melanoma survivors. J Behav Med. 2006; 29(5):419-34.

30. Moye MS, King SM, Rice ZP, DeLong LK, Seidler AM, Veledar E, Curiel-Lewandrowski C, Chen SC. Effects of total-body digital photography on cancer worry in patients with atypical mole syndrome. JAMA Dermatol. 2015; 151(2):137-43.

31. Lerman C, Trock B, Rimer BK, Jepson C, Brody D, Boyce A. Psychological side effects of breast cancer screening. Health Psychol. 1991;10(4):259-267.

32. Smith S, Fielding S, Murchie P, Johnston M, Wyke S, Powell R, et al. Reducing the time before consulting with symptoms of lung cancer: a randomised controlled trial in primary care. Br J Gen Pract. 2013;63(606):47-54. Epub 2013/01/23.

33. Smith SM, Murchie P, Devereux G, Johnston M, Lee AJ, Macleod U, et al. Developing a complex intervention to reduce time to presentation with symptoms of lung cancer. Br J Gen Pract. 2012;62(602):e605-15. Epub 2012/09/06.

34. Zigmond AS, Snaith RP. The hospital anxiety and depression scale. ACTA Psychiatr Scand 1983; 67:361-70.

35. Ware, J, Kosinski M, Keller SD. A 12-Item Short-Form Health Survey: construction of scales and preliminary tests of reliability and validity. Med Care 1996; 34(3):220-233.

36. Lyratzopoulos G, Saunders CL, Abel GA, McPhail S, Neal RD, Wardle J, Rubin GP. The relative length of the patient and the primary care interval in patients with 28 common and rarer cancers. Br J Cancer 2015; 112 Suppl:S35-40.

For peer review only

**Table 1: Piloting the intervention- consumer characteristics**

| | Participants (n=16) |
|---|---|
| ***Risk of Melanoma*** | |
| High risk of Melanoma (treated melanoma) | 8 |
| At above population risk | 5 |
| Unknown risk (relative of high risk participant) | 3 |
| ***Gender*** | |
| Male | 8 |
| Female | 8 |
| ***Age*** | |
| <35 years | 2 |
| 36-44 years | 1 |
| 45-54 years | 3 |
| 55-64 years | 7 |
| 65-74 years | 3 |
| ***Ethnicity*** | |
| White British | 15 |
| White Other | 1 |
| ***Education (highest qualification)*** | |
| GCSE or equivalent* | 1 |
| A Level or equivalent** | 3 |
| Vocational | 2 |
| Undergraduate degree | 3 |
| Postgraduate degree or professional qualification | 7 |
| ***Employment status*** | |
| Student | 1 |
| Work part-time | 5 |
| Work full-time | 4 |
| Homemaker | 1 |
| Retired | 5 |
| ***Index of Multiple Deprivation (IMD) Quintiles*** | |
| Least deprived 1 | 8 |
| 2 | 5 |
| 3 | 2 |
| 4 | 0 |
| Most deprived 5 | 0 |
| Unknown | 1 |

* GCSE- General Certificate of Secondary Education, an academic qualification awarded in a specified subject, generally taken in a number of subjects by pupils aged 14-16 in England & Wales.
** A level, same, generally taken aged 16-18.

For peer review only



For peer review only - http://bmjopen.bmj.com/site/about/guidelines.xhtml

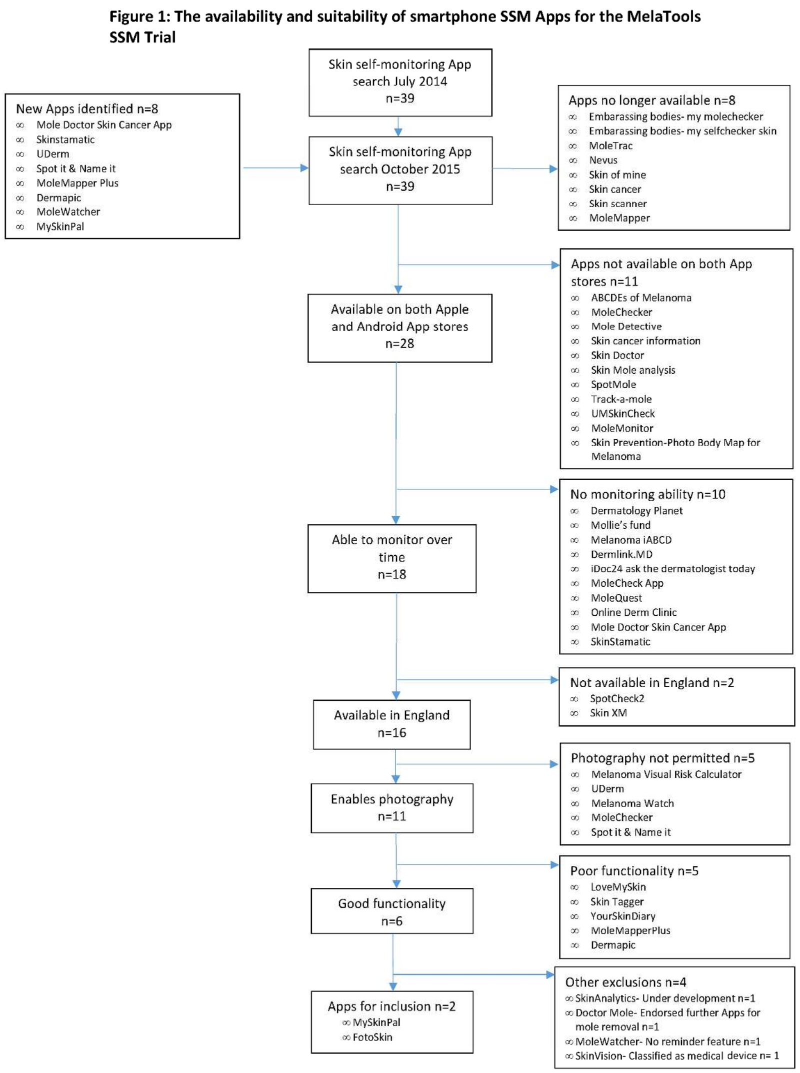


Figure 1: The availability and suitability of smartphone SSM Apps for the MelaTools SSM Trial

209x297mm (300 x 300 DPI)

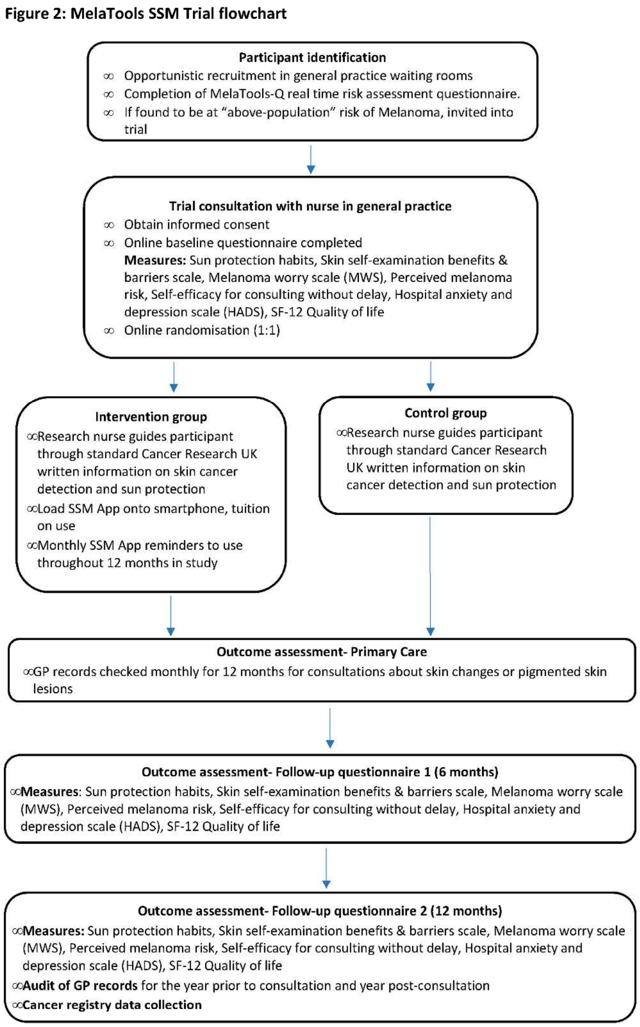


Figure 2: MelaTools SSM Trial flowchart

209x297mm (300 x 300 DPI)

UNIVERSITY OF CAMBRIDGE
Public Health and Primary Care
The Primary Care Unit

MelaTools
improving early diagnosis

NHS
National Institute for Health Research

# MelaTools Skin Self-Monitoring study for primary care patients at higher risk of melanoma

## Consent Form

**Names of Researchers**: Fiona Walter, Katie Mills

Please initial **each** box

1. I confirm that I have read and understand the Participant Information Sheet Version 2, dated 24 June 2016 for the above study and I confirm that the study procedures and information have been explained to me. I have had the opportunity to ask questions and I am satisfied with the answers and explanations provided. ☐
2. I understand that my participation in this study is voluntary and that I am free to withdraw at any time, without giving a reason and without my medical care or legal rights being affected. ☐
3. I understand that sections of my medical notes or information related directly to my participation in this study may be looked at by responsible individuals from the sponsor, regulatory authorities and research personnel where it is relevant to my taking part in research. I give permission for these individuals to have access to my records, including follow-up up to 10 years. ☐
4. I understand that my GP will be informed of my participation in this study and sent details of the study. No medical advice regarding my skin will be issued by the study team and it is my responsibility to contact my GP if I become concerned about a skin change or mole. I understand that the study team will inform my GP if my questionnaire indicates that I am more worried about my health than others. ☐
5. I understand that the researchers in charge of this study may close the study, or stop my participation in it at any time without my consent. ☐
6. I have read and understood my responsibilities for the study. ☐
7. If I am in the group that is invited to download the smartphone App, I understand that the App is accessible to the public on online App stores and is installed onto my own device at my own risk. The study team does not take responsibility for any affect that the App may have on my device or for damage to my device whilst using the App. I am welcome to delete the App from my device at any time. ☐
8. If I am in the group that is invited to download the smartphone App, I understand that the information collected on the App is for my personal use only and the study team will never ask to see this information. ☐
9. I agree to participate in this study. ☐

| Title | Name of participant | Signature | Date |
|---|---|---|---|
| Title | Name of person taking consent | Signature | Date |

*Thank you for completing this form. White copy for research team; yellow copy for participant, blue copy for GP practice.*

For peer review only - http://bmjopen.bmj.com/site/about/guidelines.xhtml

SPIRIT
STANDARD PROTOCOL ITEMS: RECOMMENDATIONS FOR INTERVENTIONAL TRIALS

SPIRIT 2013 Checklist: Recommended items to address in a clinical trial protocol and related documents*

| Section/item | Item No | Description | Addressed on page number |
|---|---|---|---|
| **Administrative information** | | | |
| Title | 1 | Descriptive title identifying the study design, population, interventions, and, if applicable, trial acronym | ________1______ |
| Trial registration | 2a | Trial identifier and registry name. If not yet registered, name of intended registry | ________2_____ |
| | 2b | All items from the World Health Organization Trial Registration Data Set | ________n/a____ |
| Protocol version | 3 | Date and version identifier | ________12_____ |
| Funding | 4 | Sources and types of financial, material, and other support | ________12_____ |
| Roles and responsibilities | 5a | Names, affiliations, and roles of protocol contributors | _____1,11______ |
| | 5b | Name and contact information for the trial sponsor | ______11_____ |
| | 5c | Role of study sponsor and funders, if any, in study design; collection, management, analysis, and interpretation of data; writing of the report; and the decision to submit the report for publication, including whether they will have ultimate authority over any of these activities | ______12______ |
| | 5d | Composition, roles, and responsibilities of the coordinating centre, steering committee, endpoint adjudication committee, data management team, and other individuals or groups overseeing the trial, if applicable (see Item 21a for data monitoring committee) | ______12_______ |



For peer review only

For peer review only - http://bmjopen.bmj.com/site/about/guidelines.xhtml

**Introduction**

| | | | |
|---|---|---|---|
| Background and rationale | 6a | Description of research question and justification for undertaking the trial, including summary of relevant studies (published and unpublished) examining benefits and harms for each intervention | ______4-6____ |
| | 6b | Explanation for choice of comparators | ______n/a______ |
| Objectives | 7 | Specific objectives or hypotheses | ______6______ |
| Trial design | 8 | Description of trial design including type of trial (eg, parallel group, crossover, factorial, single group), allocation ratio, and framework (eg, superiority, equivalence, noninferiority, exploratory) | ______7______ |

**Methods: Participants, interventions, and outcomes**

| | | | |
|---|---|---|---|
| Study setting | 9 | Description of study settings (eg, community clinic, academic hospital) and list of countries where data will be collected. Reference to where list of study sites can be obtained | ______7_______ |
| Eligibility criteria | 10 | Inclusion and exclusion criteria for participants. If applicable, eligibility criteria for study centres and individuals who will perform the interventions (eg, surgeons, psychotherapists) | _____7______ |
| Interventions | 11a | Interventions for each group with sufficient detail to allow replication, including how and when they will be administered | ______7_______ |
| | 11b | Criteria for discontinuing or modifying allocated interventions for a given trial participant (eg, drug dose change in response to harms, participant request, or improving/worsening disease) | ___n/a________ |
| | 11c | Strategies to improve adherence to intervention protocols, and any procedures for monitoring adherence (eg, drug tablet return, laboratory tests) | ____n/a______ |
| | 11d | Relevant concomitant care and interventions that are permitted or prohibited during the trial | _____n/a_____ |
| Outcomes | 12 | Primary, secondary, and other outcomes, including the specific measurement variable (eg, systolic blood pressure), analysis metric (eg, change from baseline, final value, time to event), method of aggregation (eg, median, proportion), and time point for each outcome. Explanation of the clinical relevance of chosen efficacy and harm outcomes is strongly recommended | _______8_____ |
| Participant timeline | 13 | Time schedule of enrolment, interventions (including any run-ins and washouts), assessments, and visits for participants. A schematic diagram is highly recommended (see Figure) | ______18______ |



For peer review only - http://bmjopen.bmj.com/site/about/guidelines.xhtml

| | | | |
|---|---|---|---|
| Sample size | 14 | Estimated number of participants needed to achieve study objectives and how it was determined, including clinical and statistical assumptions supporting any sample size calculations | _____10_______ |
| Recruitment | 15 | Strategies for achieving adequate participant enrolment to reach target sample size | _____10______ |

**Methods: Assignment of interventions (for controlled trials)**

Allocation:

| | | | |
|---|---|---|---|
| Sequence generation | 16a | Method of generating the allocation sequence (eg, computer-generated random numbers), and list of any factors for stratification. To reduce predictability of a random sequence, details of any planned restriction (eg, blocking) should be provided in a separate document that is unavailable to those who enrol participants or assign interventions | _____7_______ |
| Allocation concealment mechanism | 16b | Mechanism of implementing the allocation sequence (eg, central telephone; sequentially numbered, opaque, sealed envelopes), describing any steps to conceal the sequence until interventions are assigned | _____n/a______ |
| Implementation | 16c | Who will generate the allocation sequence, who will enrol participants, and who will assign participants to interventions | ______7______ |
| Blinding (masking) | 17a | Who will be blinded after assignment to interventions (eg, trial participants, care providers, outcome assessors, data analysts), and how | ____n/a______ |
| | 17b | If blinded, circumstances under which unblinding is permissible, and procedure for revealing a participant's allocated intervention during the trial | _______n/a___ |

**Methods: Data collection, management, and analysis**

| | | | |
|---|---|---|---|
| Data collection methods | 18a | Plans for assessment and collection of outcome, baseline, and other trial data, including any related processes to promote data quality (eg, duplicate measurements, training of assessors) and a description of study instruments (eg, questionnaires, laboratory tests) along with their reliability and validity, if known. Reference to where data collection forms can be found, if not in the protocol | ______8-9____ |
| | 18b | Plans to promote participant retention and complete follow-up, including list of any outcome data to be collected for participants who discontinue or deviate from intervention protocols | ____9________ |



For peer review only - http://bmjopen.bmj.com/site/about/guidelines.xhtml

| | | | |
|---|---|---|---|
| Data management | 19 | Plans for data entry, coding, security, and storage, including any related processes to promote data quality (eg, double data entry; range checks for data values). Reference to where details of data management procedures can be found, if not in the protocol | ________10____ |
| Statistical methods | 20a | Statistical methods for analysing primary and secondary outcomes. Reference to where other details of the statistical analysis plan can be found, if not in the protocol | ___9_________ |
| | 20b | Methods for any additional analyses (eg, subgroup and adjusted analyses) | ______10____ |
| | 20c | Definition of analysis population relating to protocol non-adherence (eg, as randomised analysis), and any statistical methods to handle missing data (eg, multiple imputation) | ___10______ |
| **Methods: Monitoring** | | | |
| Data monitoring | 21a | Composition of data monitoring committee (DMC); summary of its role and reporting structure; statement of whether it is independent from the sponsor and competing interests; and reference to where further details about its charter can be found, if not in the protocol. Alternatively, an explanation of why a DMC is not needed | ___10_______ |
| | 21b | Description of any interim analyses and stopping guidelines, including who will have access to these interim results and make the final decision to terminate the trial | _____n/a_____ |
| Harms | 22 | Plans for collecting, assessing, reporting, and managing solicited and spontaneously reported adverse events and other unintended effects of trial interventions or trial conduct | ______n/a_____ |
| Auditing | 23 | Frequency and procedures for auditing trial conduct, if any, and whether the process will be independent from investigators and the sponsor | _____n/a_____ |
| **Ethics and dissemination** | | | |
| Research ethics approval | 24 | Plans for seeking research ethics committee/institutional review board (REC/IRB) approval | __11________ |
| Protocol amendments | 25 | Plans for communicating important protocol modifications (eg, changes to eligibility criteria, outcomes, analyses) to relevant parties (eg, investigators, REC/IRBs, trial participants, trial registries, journals, regulators) | ___11______ |



For peer review only - http://bmjopen.bmj.com/site/about/guidelines.xhtml

| | | | |
|---|---|---|---|
| Consent or assent | 26a | Who will obtain informed consent or assent from potential trial participants or authorised surrogates, and how (see Item 32) | ______6______ |
| | 26b | Additional consent provisions for collection and use of participant data and biological specimens in ancillary studies, if applicable | ___n/a______ |
| Confidentiality | 27 | How personal information about potential and enrolled participants will be collected, shared, and maintained in order to protect confidentiality before, during, and after the trial | _____8_______ |
| Declaration of interests | 28 | Financial and other competing interests for principal investigators for the overall trial and each study site | ___11________ |
| Access to data | 29 | Statement of who will have access to the final trial dataset, and disclosure of contractual agreements that limit such access for investigators | _____12_____ |
| Ancillary and post-trial care | 30 | Provisions, if any, for ancillary and post-trial care, and for compensation to those who suffer harm from trial participation | ___n/a_______ |
| Dissemination policy | 31a | Plans for investigators and sponsor to communicate trial results to participants, healthcare professionals, the public, and other relevant groups (eg, via publication, reporting in results databases, or other data sharing arrangements), including any publication restrictions | ___11_______ |
| | 31b | Authorship eligibility guidelines and any intended use of professional writers | ___12_______ |
| | 31c | Plans, if any, for granting public access to the full protocol, participant-level dataset, and statistical code | ___n/a______ |
| **Appendices** | | | |
| Informed consent materials | 32 | Model consent form and other related documentation given to participants and authorised surrogates | _supplementary file 1_____ |
| Biological specimens | 33 | Plans for collection, laboratory evaluation, and storage of biological specimens for genetic or molecular analysis in the current trial and for future use in ancillary studies, if applicable | ___n/a_______ |

*It is strongly recommended that this checklist be read in conjunction with the SPIRIT 2013 Explanation & Elaboration for important clarification on the items. Amendments to the protocol should be tracked and dated. The SPIRIT checklist is copyrighted by the SPIRIT Group under the Creative Commons "Attribution-NonCommercial-NoDerivs 3.0 Unported" license.



For peer review only - http://bmjopen.bmj.com/site/about/guidelines.xhtml
